# Genetic Structure and Molecular Mechanisms Underlying the Formation of Tassel, Anther, and Pollen in the Male Inflorescence of Maize (*Zea mays* L.)

**DOI:** 10.3390/cells11111753

**Published:** 2022-05-26

**Authors:** Yanbo Wang, Jianxi Bao, Xun Wei, Suowei Wu, Chaowei Fang, Ziwen Li, Yuchen Qi, Yuexin Gao, Zhenying Dong, Xiangyuan Wan

**Affiliations:** 1 Zhongzhi International Institute of Agricultural Biosciences, Shunde Graduate School, Research Center of Biology and Agriculture, University of Science and Technology Beijing, Beijing 100024, China; b20180389@xs.ustb.edu.cn (Y.W.); baojianxi@xs.ustb.edu.cn (J.B.); weixun@ustb.edu.cn (X.W.); suoweiwu@ustb.edu.cn (S.W.); b20190392@xs.ustb.edu.cn (C.F.); m202110892@xs.ustb.edu.cn (Y.Q.); m202120902@xs.ustb.edu.cn (Y.G.); 2 Beijing Engineering Laboratory of Main Crop Bio-Tech Breeding, Beijing International Science and Technology Cooperation Base of Bio-Tech Breeding, Beijing Solidwill Sci-Tech Co., Ltd., Beijing 100192, China; liziwen@ustb.edu.cn

**Keywords:** maize (*Zea mays* L.), tassel morphology, male fertility, anther and pollen, hotspot intervals (HSIs), functional genes, maize molecular breeding

## Abstract

Maize tassel is the male reproductive organ which is located at the plant’s apex; both its morphological structure and fertility have a profound impact on maize grain yield. More than 40 functional genes regulating the complex tassel traits have been cloned up to now. However, the detailed molecular mechanisms underlying the whole process, from male inflorescence meristem initiation to tassel morphogenesis, are seldom discussed. Here, we summarize the male inflorescence developmental genes and construct a molecular regulatory network to further reveal the molecular mechanisms underlying tassel-trait formation in maize. Meanwhile, as one of the most frequently studied quantitative traits, hundreds of quantitative trait loci (QTLs) and thousands of quantitative trait nucleotides (QTNs) related to tassel morphology have been identified so far. To reveal the genetic structure of tassel traits, we constructed a consensus physical map for tassel traits by summarizing the genetic studies conducted over the past 20 years, and identified 97 hotspot intervals (HSIs) that can be repeatedly mapped in different labs, which will be helpful for marker-assisted selection (MAS) in improving maize yield as well as for providing theoretical guidance in the subsequent identification of the functional genes modulating tassel morphology. In addition, maize is one of the most successful crops in utilizing heterosis; mining of the genic male sterility (GMS) genes is crucial in developing biotechnology-based male-sterility (BMS) systems for seed production and hybrid breeding. In maize, more than 30 GMS genes have been isolated and characterized, and at least 15 GMS genes have been promptly validated by CRISPR/Cas9 mutagenesis within the past two years. We thus summarize the maize GMS genes and further update the molecular regulatory networks underlying male fertility in maize. Taken together, the identified HSIs, genes and molecular mechanisms underlying tassel morphological structure and male fertility are useful for guiding the subsequent cloning of functional genes and for molecular design breeding in maize. Finally, the strategies concerning efficient and rapid isolation of genes controlling tassel morphological structure and male fertility and their application in maize molecular breeding are also discussed.

## 1. Introduction

Maize (*Zea mays* L.) is an important staple crop which has been widely used as food, animal feed and industrial raw materials [1]. According to FAOSTAT (https://www.fao.org/faostat, accessed on 16 February 2022), maize has been the second most cultivated crop after wheat, but its total production and grain yield per unit area are highest among the main cereal crops, such as wheat and rice. Worldwide, 1162.4 million tons (Mt) of maize was produced in 2020, with an average productivity of 5.75 t/ha (FAOSTAT, 2020). However, the demand for maize in the developing world is increasing [2] and there is still an urgent need to improve maize yield.

**Figure 1:**
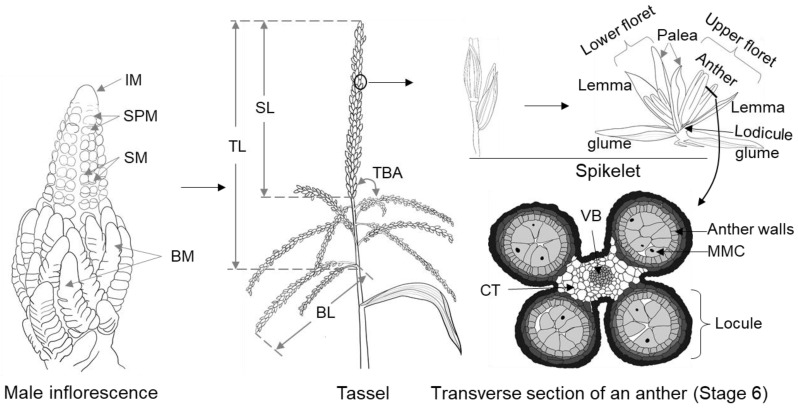
Schematic diagram of the developmental process of male inflorescence. BL: branch length; BM: branch meristem; CT: connective tissue; IM: inflorescence meristem; MMC: microspore mother cell; SL: central spike length; SM: spikelet meristem; SPM: spikelet-pair meristems; TBA: tassel branch angle; TL: tassel length; VB: vascular bundle.

Maize belongs to the Poaceae family, which contains many important crops such as sorghum, wheat and rice [3]. In contrast to these crop species, maize is monoecious with two types of unisexual inflorescences, i.e., the terminal staminate inflorescence (called tassel), which directly derives from the shoot apical meristem (SAM) that formed during embryogenesis, and the lateral pistillate inflorescence (called ear), formed in the axil of one or more leaves [4]. In fact, maize flowers are initially bisexual in the floral organ stage, with degeneration of the pistil primordia in tassel florets and staminal primordia in ear florets occurring thereafter [5,6]. As a result, the tassel and ear become male and female inflorescences, respectively [5,6]. The mature tassel consists of several basal branches and a central spike, which together bear the spikelets with two florets. The complex morphology of the mature tassel is usually features a number of sub-traits, the most researched of which are tassel length (TL), central spike length (SL), tassel branch angle (TBA), branch length (BL), tassel weight (TW) and tassel branch number (TBN) (Figure 1) [7,8,9].

Several studies have indicated that maize was domesticated from teosinte (*Z. mays* subsp. *parviglumis*) in the Balsas region of southwest Mexico about 9000 years ago [10,11]. Multiple morphological differences have accumulated in maize after domestication [12,13], such as the tassel traits, and it is now one of the most important morphological characteristics for taxonomical comparisons between them [14]. The most evident difference in tassel traits is that the tassels of cultivated maize usually possess fewer branches, whereas the tassels of teosinte are highly branched and have more than 50 branches [9]. A larger male inflorescence would decrease the photosynthetic efficiency because of the shading effect on upper leaves and it would also compete for photosynthates with the ear, so the size of the tassel is thought to be negatively correlated with the biomass and grain yield of maize [15]. Consequently, the tassel size tends to be reduced during the process of domestication and selection [16]. For example, a significant decrease in male inflorescent-related traits, such as TBN, from landraces to temperate-adapted lines was observed [16]. Duvick and Cassman [17] found that the TW of Pioneer hybrids decreased about 36% from 1967 to 1991. By examining 350 inbred lines from multiple eras of germplasm in both the USA and China, Wang et al. [18] revealed that breeders preferred to select varieties with smaller tassel size, which showed a reduction in TBN. Maize is one of the most successful crops in utilizing heterosis, and the hybrids are widely used in maize production. This reduction is possibly the result of selecting hybrids with moderately small tassels for enhancing maize grain yield [19]. However, tassels will become very small if this trend continues, eventually resulting in a difficulty maintaining and propagating maize inbred lines for hybrid seed production. In practice, breeders usually select male parents with a relatively larger tassel and plenty of pollen grains released over a desired period of time, and female parents with a smaller tassel and a relatively larger ear so that more energy is used for kernel development [8].

Tassel traits, such as TL, SL, TBA, TW, TBN and BL, are typically controlled by multiple genes [7,8,9]. Optimizing the morphological structure of the tassel via genetic selection to improve grain yield is challenging. Fortunately, much progress has been obtained through traditional quantitative trait loci (QTL) mapping and classical mutant analysis by long-term efforts [8,20,21,22,23,24]. Recently, with the fast progress of next-generation sequencing (NGS) technologies, genome-wide association studies (GWAS) have also been rapidly used for decoding the genetic basis of tassel-related traits in maize [9,18,25,26,27]. Consequently, dozens of functional genes have been well characterized and hundreds of linked QTLs and thousands of associated single nucleotide polymorphisms (SNPs) as well as many putative candidate genes have been identified, paving the way for maize grain yield enhancement [9,18,23,24,25,26,27,28,29,30,31,32,33,34,35,36,37].

In hybrid seed production, male parents with sufficient quantities of fertile pollen grains released over a desirable period are preferred. Plant anther and pollen development is a complicated process governed by the synergistic expression of multiple genes, and any disturbance of them may result in male sterility [38]. In rice, cytoplasmic male sterility (CMS) lines caused by both nuclear and mitochondrial genes or photoperiod and thermo-sensitive genic male-sterile (P/TGMS) lines caused by nuclear genes have been widely utilized since the 1970s in China [39,40]. However, due to the inseparable inheritance of disease susceptibility and male sterility, the T-type CMS lines were no longer widely used commercially in maize hybrid seed production since the epidemic of southern corn leaf blight in USA [41]. Recently, the biotechnology-based male-sterility (BMS) systems by using genic male sterility (GMS) genes have been developed and showed great potential in grain yield enhancement by improving the efficiency of hybrid seed production and heterosis application [42,43,44]. Therefore, understanding the regulation network of anther and pollen development, deciphering the mechanisms of male sterility and creating new GMS lines are meaningful for maize breeding and production.

Plant anthers arise from the floral meristem, and studies have shown that the developmental process of maize anther and pollen are highly consistent with those of rice and *Arabidopsis* [43,45,46]. Each maize anther contains four identical locules; each locule has four layers of somatic tissues, i.e., epidermis, endothecium, middle layer and tapetum, from outermost to the innermost layer [43,45,46]. The tapetum plays many crucial functions, including secretion of nutrients to support gametophyte development and synthesis of most pollen exine components [43,45,46,47]. Consequently, many characterized GMS mutants in maize exhibited tapetal defects [43,44,48,49]. Pollen development comprises three major developmental stages, that is, sporogenesis, post-meiotic development of free microspores and microspore mitosis [50,51]. The pollen wall, which is comprised of an exine and intine, also has a complex structure [52,53]. Multiple genes participating in pollen wall development have been reported [43,44,48,49]. Transcriptome analysis showed that both anther and pollen development involve transcription of tens of thousands of genes in maize [53,54,55,56], most of which may play specific functional roles during anther and pollen development. To date, hundreds of GMS mutants have been identified and characterized and more than 30 GMS genes have been cloned in maize [43,44,48,49], which has greatly enhanced our understanding of the cellular, genetic and molecular mechanisms of plant anther and pollen development.

Some excellent reviews have summarized the molecular control of grass inflorescence development [57,58,59], the potential regulatory pathways modulating inflorescence architecture in maize [60], and specific aspects of the maize GMS gene form [43,44,48,49]. However, very few of them have focused on the genetic structure and molecular mechanisms underlying the formation of tassel, anther and pollen in the male inflorescence of maize. In this review, we summarized the existing literature concerning the cytological, molecular and genetic basis of maize tassel morphology and male fertility, and constructed the molecular networks underlying tassel morphology and male fertility for increasing maize grain yield by genetically manipulating the tassel and fertility traits.

## 2. Cellular Developmental Process of Maize Male Inflorescence

Maize inflorescences develop in a strictly organized manner and form different types of meristems. Compared with the ear, the tassel has distinctive and highly varied branching inflorescences, due to the diverse activities of its axillary meristems (AMs) that form varied lateral organs (Figure 1). The SAM is a stem cell niche located at the plant apical pole that depends on meristem activity for continuous proliferation and initiation of organs throughout plant development. Three zones of cells, the central zone (CZ), the organizing center (OC) and the peripheral zone (PZ), are recognized in the SAM and the latterly initiated meristems [61,62]. The CZ possesses multipotent stem cells that proliferate continually to supply a primitive cell population to the PZ, which is the starting location for leaf primordia and the AMs that subsequently generate vegetative branches or inflorescences. The OC is located below the CZ and regulates stem cell proliferation in the CZ as well as stem cell differentiation in the PZ.

In response to endogenous and exogenous signals, SAM converts into the inflorescence meristem (IM), beginning the stages of reproductive growth [63]. First, the base of the IM initiates varying numbers of primordia, called branch meristems (BMs), the quantity and rate of which are important factors for the morphology of the tassel. However, the initiation process is absent from the ear development. Then, the peripheral regions of BM and IM form transient spikelet-pair meristems (SPMs), which are also considered indeterminate meristems due to the presence of stem cells in SPM. Each SPM generates a pair of spikelet meristems (SMs), which continue to develop and produce a glume primordium to form glume that wraps the flower organs; then, the upper-floret meristem (UFM) and lower-floret meristem (LFM) successively initiate, which in turn develop as upper and lower florets, respectively [4,64,65]. Each FM then forms a floral organ including three anthers, two lodicules and a palea. Gynoecial development arrests in male inflorescence, and male gametes are produced within the anthers (Figure 1) [4,64,65].

**Figure 2:**
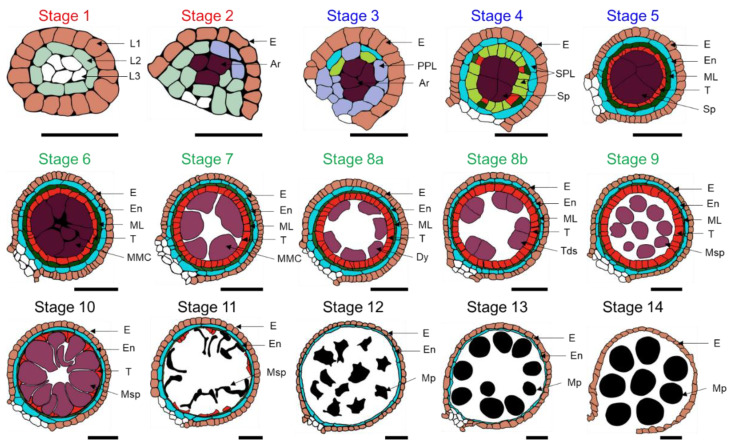
Simulated graphs of maize anther development with 14 stages covering four phases. Developmental stages belonging to phase I, II, III, and IV are labeled in red, blue, green, and black respectively. Ar: archesporial cell; Dy: dyad cell; E: epidermis; En: endothecium; L1, L2, L3: the three cell layers in stamen primordia; ML: middle layer; MMC: microspore mother cell; Mp: mature pollen; Msp: microspore; PPL: primary parietal layer; Sp: sporogenous cell; SPL: secondary parietal cell layer; T: tapetum; Tds: tetrads. Scale bars = 50 μm.

Maize anthers are comprised of the sporophyte tissue and gametophyte, which are radially symmetrical. The male gametophyte is highly specialized in maize as well as other angiosperm plants to two- or three-celled pollen grains, and its development is relatively uniform [66,67]. The connective tissue that unites the four anther locules, the vascular bundle and the anther wall together show a butterfly shape in the transverse plane and make up the sporophyte portion (Figure 1). Thus, the development and cell composition of sporophyte tissues are substantially more complicated than those of gametophytic microspores [67]. The developmental process of the maize anther is divided into 14 stages, which include four major developmental phases, i.e., phase I (archesporial (AR) cell specification), phase II (anther somatic cell division), phase III (tapetum development and pollen mother cell (PMC) formation) and phase IV (mature pollen formation and anther dehiscence) (Figure 2) [43,68]. The AR cell is the origination of male gametes in the plant germline [69]. Phase I includes anther developmental stages 1 and 2. Stamen primordium initiates and forms L1, L2 and L3 layers of cells at stage 1. Then, the formation of anther primordia: the stamen primordia differentiate to form sub-rectangular anther primordia, at which point the L2 layer of cells forms AR cells at the center of each corner. Phase II includes stages 3–5. Primary parietal cells develop and two layers of secondary parietal cells (SPL) form during stage 3 and 4. Sporogenous cells (Sp), the middle layer and the tapetum formation mainly take place at stage 5. Phase III includes stages 6–9. At this phase, microspore mother cells form a tetrad through meiosis I and II. Additionally, the middle layer becomes ribbon-like and progressively vanishes from stage 7, the cytoplasm of the tapetum is concentrated and the coloration deepens at stage 8a, and is further condensed and vacuolated at stage 8b. At stage 9, the callose outside tetrads is degenerated and microspores are released, tapetal cells become condensed and the middle layer vanishes. Phase IV includes stages 10–14. At stage 10, microspores are round and vacuolated, and tapetum degeneration occurs. At stage 11, the starch starts accumulating inside the microspore which shows falcate shape, and the tapetum has almost completely degraded into cellular debris. At stages 12 and 13, starch granules are continually accumulated into microspores, and mature pollen grains are gradually produced in the locule. Anthers release pollen grains at stage 14.

## 3. Genes and the Molecular Regulation Mechanisms of Male Inflorescence Development and Tassel Morphology

**Figure 3:**
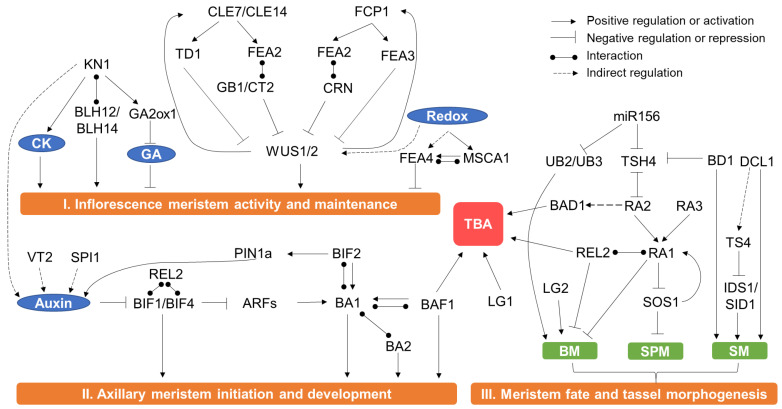
Key regulators and molecular pathways controlling male inflorescence development in maize. (**I**) Models for the roles of the CLV-WUS feedback pathway, KNOX-type proteins and FEA4 activities controlling inflorescence meristem activity and maintenance in maize. (**II**) Models of auxin biosynthesis, and signaling pathway in maize AM initiation and development. (**III**) Models of RAMOSA pathway and miRNA mediated gene regulation for inflorescence meristem fate and tassel morphogenesis. BM, branch meristem; CK, cytokinin; GA, gibberellin; SM, spikelet meristem; SPM, spikelet-pair meristem; TBA, tassel branch angle.

As mentioned above, the tassel is one of the most complex organs in maize. The processes of its initiation, development and morphology formation are well-orchestrated and controlled by multiple genes [7,9,21]. Most of the known genes controlling tassel development were identified from mutants through transposon tagging or positional cloning, which provided valuable information on how a single gene affected tassel development. Here, we summarize all the cloned genes regulating tassel development and morphology (Table 1). Furthermore, their molecular mechanisms are also proposed based on current knowledge (Figure 3).

### 3.1. Meristem Activity and Maintenance

Accurate control of plant stem cell proliferation is essential for the reproducible and continuous development of plant organs. Stem cell homeostasis is regulated by a deeply conserved negative feedback signaling between the CLAVATA (CLV) and WUSCHEL (WUS) pathways throughout plant life (Figure 3). *WUS* encodes a homeodomain transcription factor (TF), which is expressed in the OC and moves to the CZ to promote stem cell proliferation and repress differentiation, and its mutation causes a loss of SAM [116,117]. In the CLV signaling pathway, which controls stem cell differentiation, CLV3 and the related CLV3/EMBRYO-SURROUNDING REGION (CLE) members are small peptide ligands whose expressions are induced by WUS in the CZ; the peptide of CLV3 is perceived by receptor-like kinase CLV1 and related CLV1-family members or by receptor-like protein CLV2 combined with a receptor kinase CORYNE (CRN), and the signal is then transmitted to restrict the expression of WUS in OC cells [118,119,120,121]. The mutation of CLV1, CLV2 or CLV3 results in increased IM size, as well as increased numbers of floral organs [116,122]. Receptors for CLV3 have been further expanded with additional studies, such as members of the BARELY ANY MERISTEM (BAM) family and CLAVATA3 INSENSITIVE RECEPTOR KINASES (CIK) family, which indicate that the regulation of stem cell fate by CLV3 signal transduction is completed in plants [123,124,125].

The components of the CLV-WUS regulatory pathway are conservative in different plant species. *ZmWUS1* and *ZmWUS2* are predicted as the *WUS* orthologs by phylogenetic analysis in maize [77]. *Barren inflorescence3* (*Bif3*) is a dominant mutant which possesses a tandem duplicated copy of *ZmWUS1*, and its inflorescences appear defects in meristem initiation and maintenance, indicating ZmWUS1 also plays a crucial role in meristem size regulation in maize [126]. *ZmCLE7*, *ZmCLE14* and *ZmFON2-LIKE CLE PROTEIN1* (*ZmFCP1*) are CLV3 orthologs, and *FASCIATED EAR2(FEA2)* and *FEA3* are maize orthologs of *CLV2* respectively [72,127]. FEA2 can transmit signals from ZmFCP1 and ZmCLE7 through ZmCRN and the α subunit of maize heterotrimeric G protein COMPACT PLANT2 (CT2/Gα), but ZmCLE7 and ZmFCP1 transmit signals specifically via CT2 and ZmCRN, respectively [72,127]. Further information shows that G protein β subunit (ZmGB1) controls the meristem size in maize and functions together with CT2 during inflorescence development [76,128]. FEA3 transmits the ZmFCP1 signal, but genetic analysis indicates that FEA3 functions in a distinct pathway with ZmCRN and CT2 [76,128].

*THICK TASSEL DWARF1* (*TD1*) is the ortholog of *CLV1*. The *td1* mutant shows an increase in the spikelet density of maize tassel, but a synergistic phenotype appears in the *td1*;*fea2* double mutant compared to that of either single mutant, indicating that TD1 and FEA2 function in different pathways [74]. 

In addition, KNOTTED 1-Like Homeobox (KNOX) proteins also play crucial roles in meristem activity and maintenance [85]. KNOX and BELL1-like homeobox (BLH) proteins belong to the three-amino acid loop extension (TALE) homeodomain superfamily, which interacts to produce functional heterodimers [129]. Maize *KNOTTED 1* (*KN1*) is the first described KNOX gene in plants. BLH12 and BLH14 are cofactors of KN1, which interact with KN1; the *blh12*;*14* double mutant fails to maintain AMs and has highly branched tassels, which is similar to the *kn1* loss-of-function mutant [86,129]. KN1 cistrome shows that KN1 binds to thousands of loci, and the direct targets are strongly enriched for key regulators involved in the cytokinin, gibberellic acid (GA) and auxin signaling pathways [86]. KN1 can directly activate and bind to *ga2ox1*, which encodes an enzyme that inactivates GA, and the KN1 binding site of *ga2ox1* genes is also present in different gramineous plants, indicating that the local regulation of GA levels by KNOX proteins is conserved in gramineous plants [87]. However, KN1 has numerous targets that need to be further explored in order to better understand its important role in meristem maintenance.

*FEA4* is orthologous to the *Arabidopsis PERIANTHIA*, which encodes a basic region leucine zipper (bZIP) TF. The most significant phenotypes of *fea4* are a significantly thickened tassel and much higher spikelet density than the wild-type (WT) plants [73,130,131]. Phenotypic analysis of *fea4*;*fea2* double mutant suggests that FEA4 regulates meristem size in a different mechanism to the CLV-WUS pathway [73,130,131]. MALE STERILE CONVERTED ANTHER1 (MSCA1) is a maize CC-type glutaredoxin that can modify FEA4 activity through adjustment of the redox (reduction/oxidation) state and direct interaction with FEA4, thus controlling the meristem size [73,130,131]. Redox signaling also plays an important role in controlling meristem growth by regulating related genes such as *WUS* [132]. Thus, modification of the Redox signaling pathway should be a potential way to manipulate plant meristem activity.

### 3.2. Axillary Meristem Initiation and Formation in the Reproductive Phase

After the vegetative development shifts to reproductive, the radially symmetric SAM is progressively disrupted with laminar initiation of the AM, which is tightly associated with auxin accumulation in the IM peripheral zone. Auxin plays a key role in the initiation of AM (Figure 3). *VANISHING TASSEL2* (*VT2*) encodes a co-ortholog of *Arabidopsis* TRYPTOPHAN AMINOTRANSFERASE OF ARABIDOPSIS1 (TAA1), which converts tryptophan to indole-3-pyruvic acid [97]. *SPARSE INFLORESCENCE1* (*SPI1*) is a flavin monooxygenase with a similarity to *Arabidopsis* YUCCA (YUC) genes, which also work in the auxin biosynthesis pathway [91]. Loss of function of *VT2* or *SPI1* leads to defective initiation of the axillary meristem in maize [97]. Degradation of AUXIN/INDOLE-3-ACETIC ACID (Aux/IAA) proteins is a crucial step for auxin signaling in plants [133]. *BARREN INFLORESCENCE1* (*BIF1*) and *BIF4* encode Aux/IAA proteins in maize; initiation of AMs in the inflorescence of *bif1* and *bif4* mutants is inhibited, and as the result the numbers of branches and spikelets of mature tassels is largely reduced [80,81]. Upon the polar transportation of auxin to the PZ region of the SAM, BIF1 and BIF4 are degraded, which activates the expression of AUXIN RESPONSE FACTOR (ARF) TFs to promote the expression of *BARREN STALK1*(*BA1*) [81]. *BA1* encodes a bHLH TF which regulates the initiation of AMs, and the *ba1* mutant produces plants without reproductive AMs [79]. *BARREN STALK FASTIGIATE1* (*BAF1*) encodes an AT hook TF that can transcriptionally regulate *BA1* expression, and both the TBN and TBA are decreased in the *baf1* mutant [113]. *BIF2* encodes a PINOID-like serine/threonine protein kinase, which can phosphorylate BA1 and the auxin transport protein ZmPINFORMED1a (ZmPIN1a) [83,84,134]. BIF2 positively regulates auxin transport [135], and the tassel branches, spikelets, and florets are reduced in *bif2* mutants [82]. The *ba1;bif2* double mutant exhibits slight additive effects when compared to the single mutant, indicating that *BIF2* and *BA1* play overlapping and distinct roles in inflorescence development [83,84,134].

*BA2* is the co-ortholog of rice gene *LAX2,* encoding a protein that heterodimerizes and co-localizes with BA1 in the nucleus [64,136]. The *ba2* mutant is similar to *ba1*, indicating *ba2* also plays a role in reproductive AM development [64]. However, double mutant analysis showed that BA2 functions in parallel with BAF1 and BIF2 in reproductive AM development [64].

Combining the above information, auxin biosynthesis and signaling play a central role in the generation of AMs. However, genetic interaction tests for *BA1*, *BA2*, *BAF1* and *BIF2* suggest that more than one regulatory pathway may exists during this developmental process in maize, and much more efforts should be made for further elucidation of the molecular mechanism regulating AM initiation.

### 3.3. Regulation of Meristem Fate

The initiation and development of each type of meristem are tightly regulated during inflorescence development. The main IM first initiates a number of BMs that form long branches of the tassel in normal maize tassels. It was proposed that *Unbranchded2* (*UB2*) and *UB3,* which encode SQUAMOSA promoter binding (SBP)-box TFs, control the rate of axillary primordia initiation [95]. The mutation of *UB2* or *UB3* results in significant reduction in TBN [95]. Another SBP-box TF, TASSELSHEATH4 (TSH4), is also required in branch meristem initiation and maintenance; the *tsh4* mutant has reduced TBN and presents long bract leaves in place of tassel branches [93,95]. The phenotype of the *ub2;ub3* double mutant is enhanced by the loss of function of *TSH4*, indicating the functional redundancy of these three genes [93,95]. Expression analysis showed all three genes were expressed complementarily to miR156, indicating these SBP-box TFs are targets of miR156 [95].

*LIGULELESS2* (*LG2*) encodes a bZIP protein and was initially reported to regulate leaf angle by narrowing the ligular region during maize leaf primordia development [137]. In the reproductive stage, the *lg2* mutant shows a reduced number of TBN compared to its WT siblings [88]. This indicates LG2 plays an important role in the transition from the vegetative stage to the reproductive stage in the shoot apex. However, the molecular mechanism of *LG2* regulating the fate of branch meristems remains unclear. 

The branching and fate of SPM are also significantly regulated by the RAMOSA pathway [138,139], in which the known genes *RAMOSA1* (*RA1*), *RA2* and *RA3* encode different types of proteins (Figure 3). *RA1* and *RA2* encode a Cys2-His2 zinc-finger protein and a LOB-domain-containing TF, respectively [104]. *RA3* encodes a functional trehalose-6-phosphate phosphatase (TPP) and regulates inflorescence branching by mediating sugar signals [105]. The phenotypes of these mutants similarly give rise to a highly branched tassel, indicating the potential regulatory role of RAMOSA genes on meristem determinacy. Further molecular and genetic analysis show that RA1 acts downstream of both RA2 and RA3 [104,105]. Genetic analysis indicates that *TSH4* is epistatic to the three *RA* genes, and expression analysis shows TSH4 has a mutual negative regulatory relationship with RA2 but not with RA1 or RA3 [93]. Similar to *Arabidopsis* TOPLESS protein, *RAMOSA ENHANCER LOCUS2* (*REL2*) encodes a transcriptional repressor. The mutation of *REL2* enhances the phenotype of *ra1* and *ra2*, and REL2 can physically interact with RA1, but not with RA2 and RA3, which was validated by both BiFC and two yeast hybrid assays [106]. The paired spikelets in specific Andropogoneae plants, including maize, are thought to be an evolutionary trait [140]. The *Suppressor of sessile spikelets1* (*SOS1*) mutation resulted in the generation of a single spikelet in the inflorescence, and reduced TBN [140]. Although the *SOS1* gene has not been cloned yet, investigation of the interaction between *SOS1* with RAMOSA genes indicated that SOS1 functions downstream of RA1, and SOS1 may also have a positive regulatory role with RA1, which forms a negative feedback loop in controlling SPM determinacy [140]. However, despite the progress made in genetic and molecular studies of RAMOSA genes regulating the SPM fate, it is still unclear how this important pathway works.

The identity and determinacy of the SM are crucial for the biological function of the male inflorescence (Figure 1). *Branched silkless1* (*BD1*) encodes an ethylene-responsive element-binding factor (ERF) TF. In the *bd1* mutant, the identity of the spikelet meristem is altered and produces a series of spikelets indeterminately in the tassel [99]. Further genetic and expression analysis showed that BD1 negatively regulates TSH4 protein accumulation [93,95].

Plant miRNAs are endogenous short-length RNAs that post-transcriptionally repress gene expression and play crucial roles in regulating plant vegetative and reproductive developmental processes [141]. RNA endonuclease DICER-LIKE1 (DCL1) is the core component for miRNA processing during plant miRNA biogenesis; mutations of *Arabidopsis* and rice *DCL1* genes have led to obvious developmental defects [142,143]. SPMs of the *fuzzy tassel* (*fzt*) mutant produce more than two SMs and SMs produce extra FMs, which resemble the *tassel seed4* (*ts4*) mutant [110]. Positional cloning showed that *fzt* encodes a mutation in a dcl1 homolog while *ts4* is caused by a dysfunction mutation in miR172e [110,111]. Expression of miR172e is substantially reduced in *dcl1-fzt* mutants, suggesting miR172e is possibly regulated by DCL1 and that miRNAs are required for meristem determinacy. Furthermore, TS4 targets the indeterminate spikelet1 (IDS1) and the sister of indeterminate spikelet1 (SID1), both of which encode APETALA2 (AP2)-like proteins that are required for IM branching, SM determinacy and FM initiation [101,111]. 

Taken together, the determinacy of the meristem fate is a much more complicated process that involves different types of genes. However, interactions of the molecules among different pathways still remain largely unknown, and it will be interesting to test the relationship between miRNA and RAMOSA pathways, considering that mutual negative regulation between RA2 and TSH4 has been detected [105].

### 3.4. Regulation of TBA

TBA is one of the main traits in tassel morphology; a larger TBA increases the dispersed area of the pollen but affects photosynthesis because of the shading on the leaves [15]. One reason for the angle of the spikes is the presence of the pulvinus that lies between the main axis and the branch of the spikes; in addition, the degree of softness and stiffness of the spikes can cause variations in the branch angle [112,114]. The maize gene *BRANCH ANGLE DEFECTIVE* (*BAD1*) has been shown to encode a plant-specific TCP TF and to be expressed in the tips of leaf primordia, SPM, SM and husk leaves [112]. In the *bad1* mutant, the size of the pulvinus is decreased, affecting TBA. Interestingly, *RA2,* which affects the identity of SM, and *REL2,* which is the enhancer of *RA1* and *RA2* of the RAMOSA pathway, also have impact on TBA [104,106]. *BAD1* expression and protein accumulation have a large reduction in *ra2* mutant inflorescences, suggesting *RA2* functions positively upstream of *BAD1* in controlling TBA in maize. *LIGULELESS1* (*LG1*), which was reported to initially regulate the leaf angle [144], was also found to regulate TBA, whereas the expression pattern of *BAD1* has no influence on the *lg1* mutant, implying *LG1* may function parallelly with BAD1 [112]. 

BAF1 participates in AM formation, and the primary branches of *baf1* tassels are more upright and shorter compared with those of WT plants [113]. In the *baf1* mutant, a reduced pulvinus was clearly observed in the base of lateral branches, while the pulvinus of the *rel2* mutant showed an increase in lignification but nearly identical size and shape compared with WT plants, indicating different mechanisms underlying TBA formation exist in maize [106].

## 4. Genetic Basis of Tassel Traits Revealed by Quantitative Genetics Methodologies

Most of the tassel traits, including TL, SL, TBA, TW, TBN, total branch length (TBL) and others, can be quantified simply, inexpensively and precisely, making maize tassel architecture one of the systems susceptible to QTL research [8]. With recent advances in molecular biology techniques, many tassel architecture-related QTLs have been mapped in the maize genome. Berke and Rocheford [7] first reported the QTLs impacting tassel traits, including TBA, TBN and TW, and detected six, three and seven associated QTLs, respectively. Latterly, Mickelson et al. [20] identified three TBA and six TBN QTLs using a population composed by recombinant inbred lines (RILs). Upadyayula et al. [21] then detected QTLs that influence developmentally relevant patterns of tassel architecture in a BC_1_S_1_ population which derived from self-pollinated BC_1_ plants. Recently, by using 866 maize-teosinte RILs and 19,838 SNP markers, a high-resolution QTL mapping for five tassel morphological traits has been conducted, and in total 72 QTLs were identified with many known maize inflorescence development related genes significantly enriched in the mapped regions [9]. In classical linkage mapping, bi-parental segregating populations were typically used, so only a limited number of genetic loci explaining partial phenotypic variation could be identified [7,8,20,21,22,23]. The strategy of GWAS benefiting from high genetic diversity and historical accumulation of recombination events in natural populations, leading to the rapid decay of linkage disequilibrium, has been recently widely used [145]. For example, Yang et al. [29] conducted GWAS for 17 agronomic traits using 513 inbred lines, and a different number of loci were detected by the methods of the Anderson-Darling (A–D) test and mixed linear models (MLM), respectively. However, both QTL mapping and GWAS have their own merits and demerits [145,146,147]. In order to increase the accuracy of genetic mapping, a combination of GWAS and linkage mapping was recently applied to detect common quantitative trait nucleotides (QTNs) in order to uncover the genetic architecture of maize tassel traits. QTL mapping and GWAS for four tassel traits using a maize nested association mapping (NAM) population were conducted by Brown et al. [33], which identified 26–39 QTL and 241–325 SNP associations with four tassel traits. By using NAM populations from both the USA and China, and a global association panel, Wu et al. [28] dissected the genetic basis of TL and TBN, and identified a total of 125 QTLs and 965 QTNs, respectively. Wang et al. [27] evaluated TBN using 359 inbred lines and 273 doubled haploid lines; a total of 19 QTLs and 295 QTNs were identified by QTL mapping and GWAS, respectively, and one physical overlap between QTNs and QTLs was further detected.

The above QTLs/QTNs have given valuable insights into the genetic components governing maize tassel variation. Here, we summarize tassel-trait QTLs and QTNs according to the genetic studies conducted over the past 20 years, in order to construct a consensus map revealing the genetic architecture of tassel-related traits. All the positional information was normalized to the B73 reference genome version 4 (RefGen_v4.0, https://www.maizegdb.org/, accessed on 16 February 2022), and a total of 586 QTLs and 1816 QTNs for seven tassel traits were collected. The chromosomal distribution of these QTLs and QTNs was firstly examined, revealing that the number of both QTLs and QTNs on chromosome 1 is the most abundant (Figure S1), implying that chromosome 1 may contain more tassel-trait related genes. Consistent with this assumption, relatively larger numbers of genes have been cloned from chromosome 1 (Table 1). For a specific trait, TBN, for example, the number of both QTLs and QTNs on chromosome 2 is higher than on other chromosomes (Figure S1), indicating TBN-related genes are enriched on chromosome 2, or some of the genetic loci can be repeatedly identified from different populations or under different environments. The repeatedly mapped loci should be more important for further gene cloning and molecular breeding. For the QTLs, the intervals that could be mapped by three or more independent studies and QTLs with intervals <20 Mb were defined as QTL-hotspot intervals (QTL-HSIs). For the QTNs, the regions containing four or more significant SNPs in a 1 Mb interval by using the sliding window analysis method were defined as QTN-hotspot intervals (QTN-HSIs). The ability of the HSI to anchor to the same physical interval by using different genetic mapping populations and methods provides strong evidence that the HSI is important in regulating the target traits. Such an association would be more convincing if the HSI also contains known genes regulating male inflorescence development.

**Figure 4:**
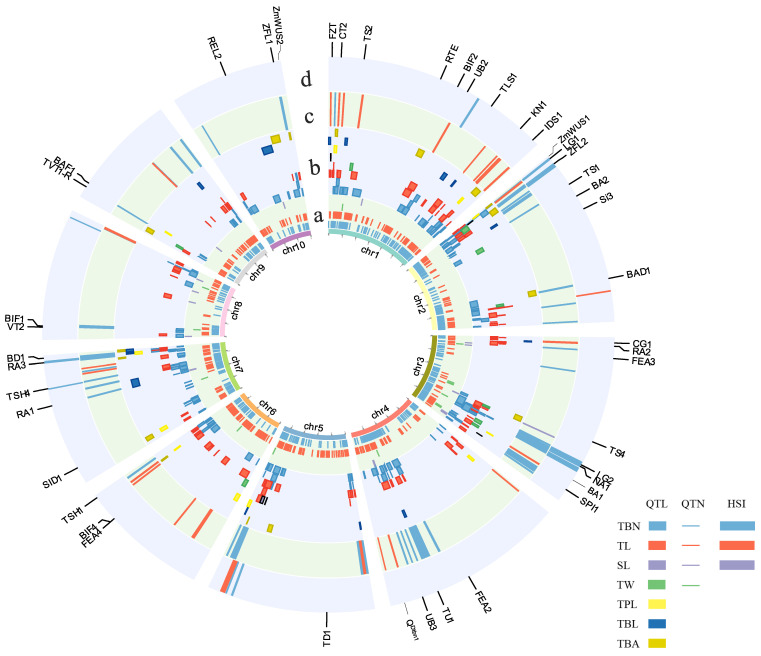
Consensus map of the genetic loci for tassel traits and distribution of the HSIs on maize chromosomes. The circle (**a**) represents the distribution of QTNs on chromosomes; circle (**b**) shows the distribution of QTLs on chromosomes; circle (**c**,**d**) represent the distribution of QTN-HSI and QTL-HSI on maize chromosomes, respectively. The physical position of known male inflorescence-related genes are shown on the outermost of the circles.

In total, we identified 18 QTL-HSIs and 79 QTN-HSIs involving three traits, i.e., SL, TBN and TL (Figure 4, Table S1). Among the 18 QTL-HSIs that are located on chromosome 1, 2, 3, 5, 7 or 8, two TL QTL-HSIs and 16 TBN QTL-HSIs were retrieved, with four HSIs containing the known male inflorescence development-related genes (*ZmWUS1*, *LG2*, *BA1* and *RA3*) (Figure 4, Table S1). TBN was significantly reduced or absent in LG2 and BA1 loss-of-function mutants [79,88], but functional deletion of *RA3* resulted in an increase in the TBN [105], indicating that the genetic loci may have opposing regulatory effects. Among the 79 QTN-HSIs, 27 TL QTN-HSIs, 51 TBN QTN-HSIs and 1 SL QTN-HSI were identified, with seven known genes (*LG1*, *ZFL2*, *BAD1*, *SPI1*, *TSH4*, *BIF1*, *ZmWUS2* and *ZFL1*) located within the TBN QTN-HSIs, and two genes (*CT2* and *UB2*) resided within the TL QTN-HSIs (Figure 4, Table S1). In summary, 14 of the HSIs possess cloned genes, and the remaining HSIs should be the important candidate regions for further male inflorescence development-related gene cloning.

Meanwhile, a total of six common HSIs (CHSIs) were found between the TBN QTN-HSIs and TBN QTL-HSIs. *LG1* and *LG2*, which are involved in inflorescence development, are located in the CHSIs chr2: 3.42–5.03 Mb and chr3: 177.34–182.12 Mb, respectively. The remaining four CHSIs are chr2: 124.06–13.07 Mb, chr2: 14.88–14.91 Mb, chr3: 186.08–188.32 Mb and chr7: 172.33–174.09 Mb. It is noteworthy that the two CHSIs possessing *LG1* or *LG2* still have the possibility of containing new candidate genes, as tassel traits have highly polygenic genetic architectures [9]. In addition, the CHSIs without known genes are possibly the most important targets for cloning new tassel-trait genes.

## 5. Application of the Quantitative Genetic Variation Regulating Tassel Traits in Maize Breeding

Many studies have shown that the majority of the identified loci have minor impacts on tassel traits, and therefore cannot be simply and directly employed in molecular breeding. However, there has been some progress in the application of the quantitative genetic variations in maize tassel-trait improvement recently. For example, one QTN (*Q^Dtbn1^*) for TBN was detected by GWAS located in the 5′-upstream of an F-box/kelch-repeat protein coding gene; the TBN was significantly reduced in F-box gene over-expression and the hybrid lines, indicating that *Q^Dtbn1^* negatively influences TBN via a dominant model, and making *Q^Dtbn1^* particularly valuable in maize hybrid breeding [89].

Fine-tuning of the inflorescence meristem-related genes provides another strategy for their utility in maize breeding. FEA2 is a core component in the CLV-WUS regulatory pathway and controls stem cell differentiation; the *fea2* mutant showed higher spikelet density in the tassel central spike and a shorter, wider and flattened ear compared with normal plants [71]. A series of weak alleles of *FEA2* were generated by ethyl methane sulfonate (EMS) mutagenesis and phenotypic analysis showed that the IMs of these *fea2* weak mutants were substantially larger than WT siblings. Further yield parameter measurement confirmed that these weak alleles of *FEA2* can increase kernel row number (KRN) without compromise in ear length or causing the fasciation that occurs in the *fea2* loss-of-function mutant, which is detrimental to yield [127]. Interestingly, a series of weak promoter alleles of *ZmCLE7* and two null alleles of *ZmCLE1E5*, which is a partially redundant compensator for *ZmCLE7*, were generated through CRISPR/Cas9 genome editing [148]. Similar to the *fea2* weak mutants, the IMs of these types of mutants were significantly larger than those of their controls [148]. Many yield-related traits, including KRN, cob diameter, ear diameter, ear weight, kernel depth and grain yield per ear were significantly increased, and notably, unlike the *Zmcle7* null allele, the ears of these mutants were not fasciated [148]. One of the important phenomena is that the tassel length was slightly affected in *Zmcle1e5* mutants [148], implying a potential for manipulating inflorescence development-related genes for tassel-trait improvement. Considering that the null mutation of most of those genes results in serious defects in tassel development, the generation of weak alleles through CRISPR-based/EMS mutagenesis or by exploitation of compensation and redundancy mechanisms should also be more applicable in tassel-trait modification [148].

## 6. The Cloned Maize GMS Genes and Their Roles in Anther and Pollen Development

Plant anther and male gamete development is a fairly complicated process, and abnormalities occurring at any stage of anther development may cause male sterility [38]. Studies have shown that male fertility in maize also has a highly polygenic genetic architecture. More than 200 maize GMS mutants have been characterized so far [149], while only 34 GMS genes were successfully cloned [150], which can be classified into four categories according to their functional properties (Table 2).

**Figure 5:**
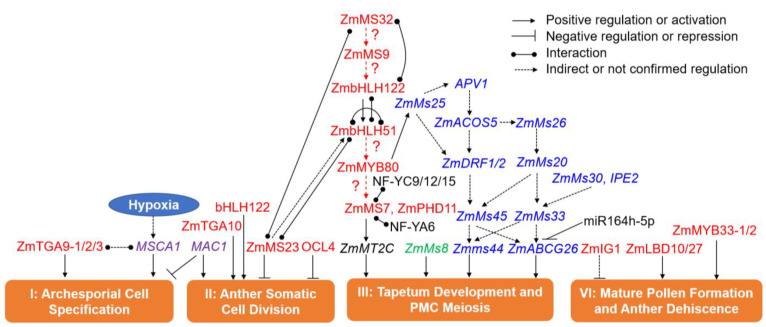
An updated working model for maize GMS genes regulating anther and pollen development. The GMS genes encoding TFs are marked in red; the GMS genes related to lipid metabolism are represented in blue fonts; the sugar metabolism-related GMS gene is labeled in green; the unclassified GMS genes are in violet; and other important genes or miRNA participating in maize male fertility regulation are in black.

It is noteworthy that 15 new GMS genes have recently been identified and validated using the CRISPR/Cas9 system [68,150,151], showing the power of gene editing technologies in maize genomics and breeding. Here, a molecular regulatory network for maize male fertility is summarized and updated by considering the functional properties and sequential expression patterns of the reported maize GMS genes (Figure 5).

**Table 2:** Summary of the known GMS genes in maize.

Categories	Gene Names	Encoded Proteins	Expression Stages	Molecular Functions	Orthologs	References
Transcription Factors	*OCL4*	HD-ZIP IV transcription factor	S5	Relates to anther wall development in early stage	-	[152]
	*ZmTGA9-1*	bZIP family transcription factor	S5	Involve in the Ubisch bodies and cuticle formation of anther wall	*AtTGA9*	[68,150,153]
	*ZmTGA9-2*		S5			
	*ZmTGA9-3*		S5–S6			
	*ZmTGA10*		S5	Controls anther dehiscence	*OsTGA10*, *AtTGA10*	[68,153,154]
	*ZmMs32*	bHLH transcription factor	S6	Controls periclinal division and tapetum differentiation	*OsUDT1*, *AtDYT1*	[155,156,157]
	*ZmbHLH122*		S5–6 and S8	Relate to the formation of anther cuticle and Ubisch bodies	*OsEAT1*	[68]
	*ZmbHLH51*		S6 to S9		*OsTDR*, *AtAMS*	[68,158]
	*ZmMs23*		S5 to S8	Controls tapetal specification and maturation	*OsTIP2*/ *bHLH142*	[159,160,161]
	*ZmMs9*	R2/R3 MYB transcription factor	S6 and S9	The control point for the entry into meiosis	*OsTDF1*, *AtTDF1*	[162,163]
	*ZmMYB33-1*	MYB transcription factor	S7 and S10	Involve in the Ubisch bodies and cuticle formation of anther wall and pollen development	*OsGAMYB*, *AtMYB33/65*	[68,164]
	*ZmMYB33-2*		S7 and S10		*OsGAMYB*, *AtMYB33/65*	
	*ZmMYB80*		S8b-S9	Involves in the Ubisch bodies formation and pollen development	*OsMYB80, AtMYB80*	[68,165,166]
	*ZmPHD11*	PHD transcription factor	S8a-S8b	Involves in the Ubisch bodies and cuticle formation of anther wall	*OsTIP3*, *AtMMD1*	[68,167,168]
	*ZmMs7*		S8b-S9	Involves in tapetum development and pollen exine formation	*OsPTC1*, *AtMs1*	[169,170]
	*IG1*	LBD transcription factor	S9	Involves in leaf and embryo sac development	*OsIG1, AS2*	[171]
	*ZmLBD27*		S7–S8b, S11–S12	Important for viable pollen formation	*AtLBD10*	[68,172]
	*ZmLBD10*		S11–S12		*AtLBD10*	
Lipid Metabolism	*ZmMs33*	Glycerol-3-phosphate acyltransferase (GPAT)	S6	Plays important roles in anther cuticle, exine and Ubisch bodies formation	*OsGPAT3*	[173,174,175]
	*ZmMs26*	Cytochrome P450 monooxygenase	S6 and S9	Regulates pollen exine formation	*OsCYP704B2*, *AtCYP704B1*	[176]
	*ZmMs10/* *APV1*	P450 subfamily protein	S6 and S9	Specifically expresses in the tapetum and involve in the hydroxylation of lauric acid	*OsCYP704B2*, *AtCYP704B1*	[177]
	*ZmMs30*	GDSL lipase	S7–S8	Regulates exine formation	-	[178]
	*Zmms44*	Lipid transfer protein	S7–8	Involves in postmeiotic tapetum secretion of proteins from tapetal cells into locule	-	[179]
	*IPE1/ZmMs20*	Putative glucose-methanolcholine oxidoreductase	S8b–S9	Controls anther cuticle and exine development	*OsNP1*	[180,181,182]
	*IPE2*	GDSL lipase	S8b–S9	Essential for anther cuticle and exine formation	-	[183]
	*ZmMs6021*/ *ZmMs25*/ *ZmFAR1*	Acyl-coenzyme A (CoA)-acyl carrier protein	S9	Affects anther cuticle and Ubisch body formation as well as microspore development	*OsDPW*, *AtMs2*	[184,185,186]
	*ZmMs45*	Strictosidine synthase	S9	Involves in pollen wall initiation after tetrad stage	*OsSTRL2*, *AtLAP3*	[42,187]
	*ZmMs2/* *ZmABCG26*	ABCG transporter	S9	Transports lipid molecules to exine	*OsABCG15*, *AtABCG26*	[185,188]
	*AmACOS5-2*	Acyl-CoA Synthetase 5	-	Play a role in sporopollenin synthesis	*OsACOS12*, *AtACOS5*	[150,189,190,191]
	*ZmDFR1*	Dihydroflavonol-4-reductase 1			*OsTKPR1*, *AtTKPR1*	[150,192,193]
	*ZmDFR2*	Dihydroflavonoid reductase 2			*OsTKPR1*, *AtTKPR1*	
Polysaccharide Metabolism	*ZmMs8*	β-1,3-galactosyltransferases	S7–S8	Affects the epidermal and tapetal cells of maize anthers and meiosis at dyad stage	*AtKNS4*	[194,195,196]
Other Processes	*ZmMs22/* *MSCA1*	Plant-specific CC-type GRX	S5	Controls the redox state and the initiation of archesporial cells	*OsMIL1*, *AtROXY1/2*	[197,198,199]
	*MAC1*	Small secreted protein	S6	Suppresses excess AR proliferation, triggers periclinal division of subepidermal cells	*OsTDL1A*	[200]

### 6.1. GMS Genes in Phase I (Archesporial Cell Specification)

The AR cell is the origination of gametes in the plant germline [69]; relatively few functional genes at this early phase of anther development were identified in maize (Figure 5; Table 2). *ZmMs22/MSCA1* encodes a CC-type glutaredoxin (GRX) and regulates the initiation of AR cells [201]. In the *msca1* mutant, AR cells fail to progress and instead differentiate into vasculature, but reductive treatments can rescue AR cell specification in *msca1* anthers, indicating that hypoxia is crucial for germ cell fate [201]. Rice *MICROSPORELESS1* (*MIL1*) and *Arabidopsis ROXY1/2* encode close orthologs of MSCA1, with the male-sterility phenotypes of their mutants similar to *msca1* [199,202]. In *Arabidopsis*, ROXY1/2 directly interacts with TGACG (TGA) motif-binding proteins TGA9 and TGA10, two bZIP TFs that are expressed throughout early anther primordia, and the *tga9*/*tga10* double mutant shows male sterility, suggesting TGA9 and TGA10 work redundantly in anther development [153]. In maize, direct interaction between ZmMs22/MSCA1 and TGA type TF FEA4 was verified, but whether ZmMs22/MSCA1 interacts with the TGA9 and TGA10 orthologs has not been confirmed. Three homologs of *TGA9*, *ZmTGA9-1*, *-2*, and *-3*, were found in the maize genome, and all of them are expressed in early anther primordia [68]. The CRISPR/Cas9-mediated gene mutagenesis verified that neither of the single or double-gene mutants show male sterility; only the *ZmTGA9-1/2/3* triple-gene mutants lose the male sterility and fail to exude pollen grains, indicating the functional redundancy of the three genes in maize [68].

### 6.2. GMS Genes in Phase II (Anther Somatic Cell Division)

At maize anther developmental phase II, at least five GMS genes patriciate in anther somatic cell division (Figure 5), i.e., *MULTIPLE ARCHESPORIAL CELLES 1* (*MAC1*), *OUTER CELL LAYER 4* (*OCL4*), *ZmMs23*, *ZmbHLH122* and *ZmTGA10*. *MAC1* encodes a small secreted protein, which is possibly preferentially expressed in AR cells [200]. The proliferation of AR cells in the *mac1* mutant is more rapid than WT, and as a result, the *mac1* mutant displays extra AR cells in anthers and is completely sterile [200]. *OCL4* encodes a HD-ZIP IV TF and is specifically expressed in young organ primordia and the epidermis of shoot meristems [152]. The *ocl4* mutant shows partial male sterility, and its anthers exhibit an additional cell layer with endothecium cellular characteristics, which is probably the cause of the poor anthesis and dehiscence observed at maturity [152]. Analysis of the *mac1/ocl4* double mutant demonstrates that OCL4 and MAC1 act parallelly in controlling the proliferation of subepidermal cells [200]. The maize TGA10 ortholog ZmTGA10 expresses before stage 6 during anther development; the pollen of knockout mutants of *ZmTGA10* by CRISPR/Cas9 technology look nearly normal, but the anthers fail to dehisce [68]. Both *ZmMs23* and *ZmbHLH122* encode bHLH TFs, and play an important role in the differentiation of anther somatic wall layers [68,155,159]. In *Zmms23* mutant anthers, the tapetum divides into two layers, forming an anther with five wall layers [155,159,203]. The *Zmbhlh122* mutant displays complete male sterility, similar to *Zmms23*, but whether the defects of the tapetum happen during anther wall development has not yet been determined [68]. 

### 6.3. GMS Genes in Phase III (Tapetum Development and PMC Formation)

The tapetum, which occupies the innermost layer of the anther wall and surrounds the developing microspores, plays sequential and essential roles in ferrying nutrients to microspores, remodeling the extracellular callose covering the PMCs or tetrads, and secreting exine precursors onto maturing pollen grains [155,159], so that mutation of many GMS genes exhibit tapetal defects [149]. Over half of the cloned maize GMS genes function mainly in this phase, and a relatively complex molecular network, involving transcription regulation, lipid and sugar metabolism and others, was revealed (Figure 5).

In *Arabidopsis*, a DYT1-TDF1-AMS-MS188-MS1 regulatory network controlling tapetum development and pollen formation has been validated [162,204]. Although the corresponding TFs (ZmMS32, ZmMS9, ZmbHLH51, ZmMYB80 and ZmMS7) are found to be encoded by GMS genes, whether a similar genetic pathway and regulatory network exist in maize needs to be investigated. Nan et al. [68,150,151] compared the mutant phenotypes of four *bHLH* genes, i.e., *ZmMs32*, *ZmbHLH51*, *ZmMs23* and *ZmbHLH122*, and found that all four *bhlh* mutants have an aberrant tapetum. Combining several recent studies, a more complicated cascade of bHLH-regulated pathways was established (Figure 5) [68,151,155,159]. Protein-protein interaction assays showed that ZmMS23 interacts with both ZmMS32 and ZmbHLH51, ZmbHLH122 interacts with both ZmMS32 and ZmbHLH51, and ZmbHLH51 forms homodimers. Supported further by cytological observation and gene expression analysis in each mutant, the order of essential actions is ZmMS23, ZmMS32, ZmbHLH122, and then ZmbHLH51 [68,151,155,159]. ZmbHLH122 directly activates *ZmbHLH51* and interacts with ZmbHLH51 [68], but it appears that *ZmbHLH122* is not a direct target of ZmMS23 or ZmMS32 [155,159]. 

Maize *ZmMs9* is the homolog of *Arabidopsis TAPETAL DEVELOPMENT AND FUNCTION 1* (*TDF1*) [162]. In *Arabidopsis*, TDF1 (a MYB TF) works downstream of DYSFUNCTIONAL TAPETUM 1 (DYT1) (a bHLH TF), the ortholog of ZmMS32 [205], and bHLH proteins interact with DYT1 and act downstream of TDF1 to activate the target genes through feed-forward and positive feedback loops [206,207]. We thus propose that ZmMS9 works between ZmMS32 and ZmbHLH122 (Figure 5); nevertheless, this needs to be confirmed via experimental data. ZmMYB80 is the ortholog of AtMYB80 [208]; the regulatory relationship between ZmMYB80 and ZmMS7 has not been validated, but mutation of each gene leads to male sterility in maize [68,169,170]. *ZmMs7* and *ZmPHD11* encode PHD-finger TFs, and the former regulates genes involved in exine formation and tapetum development as a transcriptional activator [68,169,170]. ZmMS7 can directly interact with nuclear factor Y (NF-Y) subunits NF-YA6 and NF-YC9/12/15, and then the complex directly activates expression of *ZmMT2C*, the homolog of *OsMT2b*, both of which are involved in the tapetal cell programmed cell death (PCD) of rice and maize anthers [68,169,170,209].

The second major group of GMS genes in phase III involves lipid metabolism (Figure 5; Table 2). Pollen exine (sporopollenin and tryphine), anther subcellular organelle membranes’ wax, and cutin are mainly composed of lipidic substances, and any disturbance of lipid metabolism can result in microspore abortion and GMS [210], suggesting the importance of lipid metabolism in anther and pollen development. However, lipid biosynthesis and transport pathways involve many steps, most of which remain uncovered in maize [48,185].

Of the cloned lipid metabolism-related GMS genes in maize, *ZmMs6021*/*ZmMs25*/*ZmFAR1*, *ZmMs26*, *ZmMs10*/*APV1*, *ZmIPE1*/*ZmMs20*, *ZmMs30*, *IPE2*, *ZmACOS5*, *ZmDFR1/2* and *ZmMs45* are involved in exine sporopollenin and tryphine biosynthesis [150,176,177,178,179,180,181,183,184,185,186]. *FATTY ACYL REDUCTASE1* (*ZmFAR1*) encodes a plastid-localized fatty acyl reductase, and catalyzes the conversion of fatty acyl-coenzyme A or acyl carrier protein to a primary fatty alcohol in an NADPH-dependent way. Its mutation results in irregular pollen exine and a lack of the knitting cuticle and Ubisch bodies [184,185,186]. *ZmMs26* and *ABNORMAL POLLEN VACUOLATION1* (*APV1*) encode cytochrome P450 mono-oxygenases belonging to the subfamily CYP704B1 and CYP703A2, respectively, and the pollen exine, anther cuticle, and Ubisch bodies display defectively in their mutants [176,177]. *IRREGULAR POLLEN EXINE1* (*ZmIPE1*), encoding a glucose methanol choline oxidoreductase, is involved in oxidation of C16/C18 ω-hydroxy fatty acids, and thus regulates anther cuticle and exine formation [180]. *ZmMs30* encodes a novel GDSL lipase which prefers substrates with a short carbon chain, and the *Zmms30* mutant shows complete male sterility with a defective anther cuticle and an irregular foot layer of exine [178]. *IPE2* also encodes a GDSL lipase and affects the accumulation of C16/C18 fatty acids and their derivatives; mutation of *IPE2* also results in complete male sterility, which is possibly caused by the delayed degeneration of the tapetum and middle layer, and defect in anther cuticle and exine formation [183]. Maize acyl-CoA synthetase genes *ZmACOS5-1/-2* are the homologs of *AtACOS5* and *OsACOS12* and have conserved functions in sporopollenin biosynthesis, but only the *Zmacos5-2* mutant displays complete male sterility without pollen grains [150,189,191]. Maize dihydroflavonol 4-reductase genes *ZmDFR1*/*2* are the homologs of *TETRAKETIDE α-PYRONE REDUCTASE1* (*AtTKPR1*) and *OsTKPR1* [150,192,193], but *ZmDFR1*/*2* together catalyze tri- and tetra-ketide α-pyrones to produce reduced α-pyrones, since single-gene mutants show male fertility [150,192]. An atypical strictosidine synthase gene *ZmMs45* participates in the phenylpropanoid pathway that involves in proper exine formation, and the *Zmms45* mutant shows complete male sterility [185,187]. 

The anther cutin and wax biosynthetic pathways share some common steps and functional genes with the sporopollenin biosynthetic pathway [48,185]. In addition, some proteins such as *sn*-2 Glycerol-3-Phosphate Acyltransferase (GPAT) are mainly involved in anther cutin and wax biosynthesis [48,185]. *ZmMs33/ZmGPAT6* catalyzes the biosynthesis of glycolipids and phospholipids at the first step of the glycerolipid synthetic pathway [48,175]. The *Zmms33* mutant displays an abnormal anther cuticle and endothecium chloroplast, in which starch granules are continuously filled at early developmental stages [175].

*Zmms44* encodes a lipid transfer protein (LTP), which transport lipidic precursors for anther cuticle and exine formation; variation at the signal peptide cleavage site disables the secretion ability of Zmms44 protein from tapetal cells into the locule, resulting in dominant male sterility [179]. *ZmMs2*/*ZmABCG26* encodes an ATP-binding cassette (ABC) transporter involved in the transport of sporopollenin and/or tryphine [185,187]. The ZmMS2/ZmABCG26 protein is localized in the plasma membrane, chloroplast/plastid and ER. The anthers of its mutants are shrunken without viable pollen grain produced [185,187].

There is possibly a multidimensional regulatory network among different types of GMS genes involving maize anther and pollen development. By examining the differentially expressed (DE) genes in *ms23*, *ms32*, *bhlh51* and *bhlh122* mutants, many lipid metabolism-related GMS genes, including *ZmMs25*, *ZmMs26*, *ZmMs30*, *ZmMs33* and *Zmms44*, are DE in one or more of these mutants [151]. In addition, dozens of potential sugar metabolism-related GMS genes are predicted in the maize genome through bioinformatic analysis [49], but only one GMS gene, *ZmMs8*, has been cloned [185,187]. *ZmMs8* encodes 1,3-galactosyltransferase in maize, and is DE in bHLH TF mutants [151]. Thus, the lipid and sugar metabolism-related DE genes are possibly regulated by bHLH TFs. Similarly, the MYB TF ZmMYB80 directly regulates expression of *ZmMs25* (Figure 5) [184,185,186]. However, the more detailed regulatory relationship between GMS TFs and lipid and sugar metabolism-related GMS genes needs to be further investigated, which is essential for the understanding of the molecular mechanism underlying maize anther and pollen formation.

Multi-types of regulatory mechanisms of miRNAs with lipid metabolism-related GMS genes or TF GMS genes have been predicted or identified [48,185,211]. For example, *ZmABCG26* is a potential target of zma-miR164h-5p revealed by sequence analysis and expression pattern comparison between them, as well as by gene editing [185,187,211].

### 6.4. GMS Genes in Phase IV (Mature Pollen Formation and Anther Dehiscence)

Some TFs have been reported to work in this developmental phase [68]. *ZmMYB33-1*/*-2* encode MYB family TFs and act redundantly, as only the double-gene mutant generated by CRISPR/Cas9 technology exhibits complete male sterility [68]. INDETERMINATE GAMETOPHYTE1 (*IG1*), *ZmLBD10* and *ZmLBD27* encode LOB domain family TFs, and the *ig1* mutant shows sporophytic male sterility, failing to shed pollen or extrude anthers [68,171]. However, ZmLBD10 and ZmLBD27 function redundantly, as viable pollen formation was only significantly affected by their combination in maize [68,171].

It is noteworthy that the functional stages of GMS genes in regulating anther and pollen development are not always completely consistent with their expression patterns; some GMS genes may function later than their peak expression stage, while others that act at previous stages may also function at later stages, particularly with two expression peaks [48,185]. 

Taken together, transcriptional regulation, lipid and sugar metabolism and redox homeostasis have been demonstrated to be involved in maize anther and pollen development (Figure 5), and much progress in the cognizance of maize anther and pollen development has been made by dissecting the GMS mutants. Accordingly, we updated the molecular regulatory networks (Figure 5) governing male fertility in maize by combining recent discoveries with our previous work [43,48,68,150,151,170,171,175,185,186]. However, the detailed molecular mechanisms remain largely uncharacterized. More experimental data is thus required to construct an integral molecular regulatory network underlying maize anther and pollen development.

## 7. Application of GMS Genes in Maize Heterosis Utilization

The identification of more GMS genes not only boosts our understanding of molecular mechanisms of male sterility but also facilitates the development and utilization of BMS systems in hybrid seed production and the efficient application of heterosis [43]. The utility of GMS genes for developing BMS systems has been recently reported in maize. Some highly efficient hybrid seed production systems that exploit transgene technology but generate a non-transgenic final product have been developed due to the public concern about transgenic plants [43]. *ZmMs45* encodes a strictosidine synthase and its mutant shows complete male sterility [185,187]. The ‘‘Seed Production Technology’’ (SPT) using transgenic construct-driven non-transgenic seed systems was developed by DuPont Pioneer based on the *ZmMs45* restorer gene and *ms45* mutant, and the system has been commercially utilized in the USA since 2012 for maize hybrid seed production [42]. The SPT transgenic maintainer line possesses a homozygous *Zmms45* allele, and a SPT construct containing a WT *ZmMs45* gene for fertility restoration, a *ZmAA* gene to disrupt normal pollen formation and a *DsRed2* gene for seed sorting (Table 3). Consequently, the produced pollen grains of maintainer line do not carry the SPT transgenes, and half of the self-pollinated seeds are transgenic but can be separated depending on the fluorescence of the DsRed2 protein by mechanical color sorting [42].

However, transgene transmission through pollen in the SPT maintainer transformants was reported up to 0.518% [42]. New technologies are needed to further decrease the rate of transgene transmission. To this end, the Multi-Control Sterility (MCS) system, by transforming a single MCS construct into maize *ms7-6007*, *ms30-6028* or *ms33-6038* GMS mutants, was developed in our laboratory [169,174,178]. Compared with the SPT, two additional functional modules, the *Dam* and *Bar* genes, were appended to the MCS construct (Table 3); however, the transgene transmission rate was greatly decreased to lower the risk of transgene flow in commercial maize hybrid seed production [169,174,178].

Dominant genic male-sterility (DGMS) systems have been also developed and tested for seed production and hybrid breeding in maize [44]. *Zmms44* encodes a LTP and *ZmMs44* is a dominant GMS mutant; the *Zmms44-*based DGMS system was created by expressing an artificial miRNA that silences *Zmms44* expression [179]. Most importantly, the kernels per ear of *ZmMs44* sterile plants increased at an average of 9.6% compared with WT plants, due to the reduced tassel growth partitioning more nitrogen to the ear, and the hybrids carrying the *ZmMs44* allele showed significant yield increase when nitrogen is limited, indicating that the *Zmms44-*based DGMS system can not only reduce maize hybrid seed production costs but also provide a new strategy for increasing nitrogen use efficiency in maize [179]. Recently, a *ZmMs7* DGMS system has been developed in our laboratory, where *ZmMs7* was driven by the *p5126* promoter coupling with a red fluorescence seed-sorting module in a single construct [170]. The *p5126-ZmMs7* DGMS lines show dominant male sterility, whereas their female fertility and vegetative growth are normal [170]. Compared with other BMS systems, this DGMS system is simpler and more efficient, and can be broadly applied even for plants without applicable GMS genes [170].

## 8. Conclusions and Perspectives

Tassel morphology and male fertility, as two main aspects of male inflorescence development, are tightly correlated with maize yield. Over the past decades, more than 40 genes involving male inflorescence development have been identified. We further constructed a molecular regulatory network, and found that the CLV-WUS pathway, KN1 pathway and Redox signaling pathway play vital roles in inflorescence meristem activity and maintenance; auxin biosynthesis, transport and signaling transduction mainly participate in axillary meristem initiation and development, while RAMOSA pathway and some miRNA associated proteins regulate meristem fate and tassel morphogenesis (Figure 3). However, the precise optimization of tassel structure using these gene resources is challenging, as null alleles of many inflorescence meristem development-related genes have serious phenotypes [148]. Recently, weak alleles of CLE genes have been quickly generated by modifying the promoter sequences via CRISPR/Cas9 genome editing, which significantly improved multiple maize grain yield-related traits [148], suggesting that inflorescence meristem development related gene resources combining CRISPR/Cas9 technology have enormous potential in crop enhancement. It is interesting to generate different alleles of other male inflorescence development-related genes by CRISPR/Cas9 technology to test their breeding utilities. Considering many countries have permitted the release of gene editing crops [212], the generated elite alleles can be thus directly applied for crop improvement.

By integrating the results of genetic mapping conducted over the past 20 years, we constructed a consensus physical map for tassel traits and extracted 97 HSIs (Figure 4), which lays the foundation for subsequent candidate gene cloning, and provides valuable targets for MAS in improving maize yield via tassel morphology modification. Application of the quantitative genetic variations in *Q^Dtbn1^* locus for tassel-trait improvement has been reported [89]. However, GWAS studies aimed at the discovery of more tassel-trait QTNs will still be the main task in the future. With the fast development of DNA sequencing technologies, our capacity to dissect genomes far exceeds the ability to measure phenotypes of plant traits. Lacking precise, high-throughput and labor-saving phenotyping technologies is one of the bottlenecks for the genetic dissection of maize tassel traits. Recently, many efforts have been made to address this imbalance. A variety of imaging approaches, such as hyperspectral imaging, have been developed, and unmanned aerial vehicles (UAVs) are also applied in crop phenotyping [213,214]. Traditional QTL mapping and GWAS methods have their own merits and demerits, with newer approaches needed to deal with the demerits. Machine learning (ML) using iterations where the computer tries to discover the patterns hidden in the current data that are then used to predict future data have shown advantages in increasing predictive power, and the use of ML approaches has become common for GWAS in recent years [215]. We suggest that high-throughput phenotyping combined with deep ML should be quickly used for the genetic dissection of tassel traits. Last but not least, MAS has not been widely used in maize tassel trait improvement; functional and linked molecular markers should be developed based on the cloned genes and HSIs mined here, which will accelerate modifications of maize tassel traits.

Maize has developed a monoecious and diclinous flowering system during species differentiation, making it an ideal model for studying inflorescence development. Heterosis has been especially successfully utilized in maize breeding for decades and male fertility control has a great significance in maize production. Four types of GMS genes, including TF, lipid- and sugar-related, as well as the unclassified type, were summarized in this study (Table 2), and some GMS genes work redundantly. Such redundancy is difficult to detect by traditional mutagenesis and map-based cloning, but CRISPR/Cas9-mediated gene mutagenesis provided a new opportunity. It is worth noting that 15 of the 34 maize GMS genes were validated via CRISPR/Cas9 technology within the last two years [68,150,151,216]. We thus constructed and updated the working model regulating maize anther and pollen development (Figure 5). A large number of studies have demonstrated that the GMS genes tend to be specifically expressed in the anther [49,211,216]; thus, we should focus on the rapid isolation of anther-specific genes and then validate their functions by CRISPR/Cas9 mutagenesis, which is crucial for thoroughly unraveling the molecular regulatory network underlying maize anther and pollen development. Recently, different kinds of BMS systems in maize, including SPT, MCS and DGMS, have been established [42,43,44,169,170,174,178,179]; the newly identified GMS genes can be tested for their potential in increasing maize yield via the utility of BMS systems.

## Figures and Tables

**Table 1:** Known genes regulating male inflorescence development in maize.

Types	Gene Names	Gene IDs (*Zm00001d*)	Positions	Annotations	References
Genes regulating meristem activity	*CT2*	*027886*	Chr1: 16721214–16732176	Subunit (Gα) of a heterotrimeric GTP binding protein	[70]
	*FEA2*	*051012*	Chr4: 136764371–136769212	LRR receptor-like protein	[71]
	*FEA3*	*040130*	Chr3: 28709631–28715222	LRR receptor	[72]
	*FEA4*	*037317*	Chr6: 120722612–120728273	bZIP transcription factor	[73]
	*TD1*	*014793*	Chr5: 63455339–63461620	LRR receptor-like kinase	[74]
	*TVT1-R*	*045192*	Chr9: 15719302–15730427	Ribonucleotide reductase	[75]
	*ZmGB1*	*033422*	Chr1: 262590898–262600232	Subunit (Gβ) of a heterotrimeric GTP binding protein	[76]
	*ZmWUS1*	*001948*	Chr2: 3416796–3418004	Homeodomain transcription factor	[77]
	*ZmWUS2*	*026537*	Chr10: 147855536–147856873	Homeodomain transcription factor	[77]
Genes controlling BM development	*BA1*	*042989*	Chr3: 186013129–186016764	bHLH transcription factor	[78,79]
	*BA2*	*003897*	Chr2: 65741213–65756966	Nucleoprotein	[64]
	*BIF1*	*008749*	Chr8: 18950258–18955333	Aux/IAA protein	[80,81]
	*BIF2*	*031068*	Chr1: 175806351–175810932	Serine/threonine protein kinase	[81,82,83,84]
	*BIF4*	*037691*	Chr6: 134087331–134094170	Aux/IAA protein	[81]
	*KN1*	*033859*	Chr1: 276071835–276082742	KNOTTED1-like homeobox (KNOX) transcription factor	[85,86,87]
	*LG2*	*042777*	Chr3: 179386227–179397947	bZIP transcription factor	[88]
	*Q^Dtbn1^*	*053358*	Chr4: 227482692–227483909	Kelch repeat-containing F-box protein	[89]
	*RTE*	*030656*	Chr1: 151219729–151227321	Membrane-localized boron efflux transporter	[90]
	*SPI1*	*044069*	Chr3: 218285795–218290961	Flavin monooxygenase	[91]
	*TLS1*	*032461*	Chr1: 227447816–227454579	Boron channel protein	[92]
	*TSH4*	*020941*	Chr7: 137272100–137278639	SBP-box transcription factor	[93]
	*TU1*	*052180*	Chr4: 181855808–181865542	MADS box transcription factor	[94]
	*UB2*	*031451*	Chr1: 190381366–190388089	SBP-box transcription factor	[95]
	*UB3*	*052890*	Chr4: 203609847–203617018	SBP-box transcription factor	[95,96]
	*VT2*	*008700*	Chr8: 17393223–17400365	Grass-specific tryptophan aminotransferase	[97]
	*ZFL1*	*026231*	Chr10: 141560362–141566267	FLO/LFY homolog	[98]
	*ZFL2*	*002449*	Chr2: 12912591–12918568	FLO/LFY homolog	[98]
Genes controlling meristem fate	*BD1*	*022488*	Chr7: 178604458–178608405	ERF transcription factor	[99]
	*IDS1*	*034629*	Chr1: 298421359–298428550	AP2 transcription factor	[100,101]
	*NA1*	*042843*	Chr3: 181819965–181824489	5α-steroid reductase	[102]
	*RA1*	*020430*	Chr7: 113570910–113574437	Cys2-His2 zinc-finger transcription factor	[103]
	*RA2*	*039694*	Chr3: 12156780–12160565	LOB-domain transcription factor	[104]
	*RA3*	*022193*	Chr7: 172483459–172490694	Trehalose-6phosphate phosphatase	[105]
	*REL2*	*024523*	Chr10: 75992328–76004412	Transcriptional co-repressor similar to TOPLESS protein	[106]
	*Si3*	*004130*	Chr2: 85360621–85369941	Putative transcriptional regulator	[107]
	*SID1*	*019230*	Chr7: 23052961–23067089	AP2 transcription factor	[101]
	*TS1*	*003533*	Chr2: 47103687–47110872	Lipoxygenase	[6]
	*TS2*	*028806*	Chr1: 46953827–46958171	Short-chain alcohol dehydrogenase	[108]
MicroRNAs mediated inflorescence development	*CG1*	- *	Chr3: 7773052–7777625	MiRNA156	[109]
	*FZT*	*027412*	Chr1: 4722956–4738332	DICER-LIKE1	[110]
	*TS4*	- *	Chr3: 144916511–144920220	MiRNA172	[111]
Genes controlling tassel branch angel	*BAD1*	*005737*	Chr2: 185420859–185424689	TCP transcription factor	[112]
	*BAF1*	*045427*	Chr9: 21784850–21788875	AT hook transcription factor	[113]
	*LG1*	*002005*	Chr2: 4229354–4236035	SBP-box transcription factor	[114]
	*TSH1*	*039113*	Chr6: 170246513–170250985	GATA zinc-finger protein	[115]

* Gene IDs were not given in B73 reference genome (RefGen_v4.0) as these loci encode miRNAs.

**Table 3:** Breeding application of GMS genes in maize.

BMS System	Gene Names	Elements					References
SPT	*ZmMs45*	*pZmMs5126::ZmMs45*	*pPG47::Bt1:ZmAA*	*35SEN-pLTP2::DsRed2*			[42]
MCS	*ZmMs7*	*pZmMs7::ZmMs7*	*pPG47::Bt1:ZmAA*	*pLTP2::mCherry*	*p35S::Bar*	*pZm13::Dam*	[169,174,178]
MCS	*ZmMs30*	*PZmMs30::ZmMs30*	*pPG47::Bt1:ZmAA*	*pLTP2::mCherry*	*p35S::Bar*		[169,174,178]
MCS	*ZmMs33*	*PZmMs33::ZmMs33*	*pPG47::Bt1:ZmAA*	*pLTP2::mCherry*	*p35S::Bar*	*pZm13::Dam*	[169,174,178]
DGMS	*ZmMs7*	*PZmMs5126::ZmMs7*		*pLTP2::mCherry*	*p35S::Bar*		[170]
DGMS	*ZmMs44*	*pZmMs44::ZmMs44* *miRNA*	*pPG47::Bt1:ZmAA*	*35SEN-pLTP2::DsRed2*			[179]

SPT, MCS and DGMS, biotechnology-based male-sterility (BMS) systems; *ZmMs7*, *ZmMs30*, *ZmMs33*, *ZmMs44* and *ZmMs45*, maize fertility restoration genes; *pZmMs5126*, *pZmMs7*, *pZmMs30*, *pZmMs33* and *pZmMs44*, maize fertility restoration gene promoters; *pPG47*, polygalacturonase gene promoter; *Bt1*, Brittle-1 transit peptide; *ZmAA*, α-amylase gene; *pLTP2*, lipid transfer protein-2 gene promoter; 35SEN, cauliflower mosaic virus 35S enhancer; *DsRed2* and *mCherry*, red fluorescence genes; *p35S*, cauliflower mosaic virus 35S promoter; *Bar*, herbicide resistance gene; *pZm13*, *Zm13* gene promoter; *Dam*, DNA adenine methylase gene.

## Data Availability

All data are shown in the main manuscript and in the Supplementary Materials.

## Supplementary Materials

The following supporting information can be downloaded at: https://www.mdpi.com/article/10.3390/cells11111753/s1, Figure S1: Summary of genetic loci for tassel-related traits on each chromosome; Table S1: Summary of the QTL-HSIs and QTN-HSIs.

## References

[1] Ranum P., Peña-Rosas J.P., Garcia-Casal M.N. (2014). Global maize production, utilization, and consumption. Ann. N. Y. Acad. Sci..

[2] Rosegrant M., Ringler C., Sulser T.B., Ewing M., Palazzo A., Zhu T., Nelson G.C., Koo J., Robertson R., Msangi S. (2009). Agriculture and Food Security under Global Change: Prospects for 2025/2050.

[3] Group G., Barker N., Clark L., Davis J., Duvall M., Guala G., Hsiao C., Kellogg E., Linder H. (2002). Phylogeny and subfamilial classification of the grasses (Poaceae). Ann. Mo. Bot. Gard..

[4] Vollbrecht E., Schmidt R.J. (2009). Development of the Inflorescences.

[5] Dellaporta S.L., Calderon-Urrea A. (1994). The sex determination process in maize. Science.

[6] Acosta I.F., Laparra H., Romero S.P., Schmelz E., Hamberg M., Mottinger J.P., Moreno M.A., Dellaporta S.L. (2009). *tasselseed1* is a lipoxygenase affecting jasmonic acid signaling in sex determination of maize. Science.

[7] Berke T.G., Rocheford T.R. (1999). Quantitative trait loci for tassel traits in maize. Crop Sci..

[8] Upadyayula N., da Silva H.S., Bohn M.O., Rocheford T.R. (2006). Genetic and QTL analysis of maize tassel and ear inflorescence architecture. Theor. Appl. Genet..

[9] Xu G.H., Wang X.F., Huang C., Xu D.Y., Li D., Tian J.G., Chen Q.Y., Wang C.L., Liang Y.M., Wu Y.Y. (2017). Complex genetic architecture underlies maize tassel domestication. New Phytol..

[10] Matsuoka Y., Vigouroux Y., Goodman M.M., Sanchez G.J., Buckler E., Doebley J. (2002). A single domestication for maize shown by multilocus microsatellite genotyping. Proc. Natl. Acad. Sci. USA.

[11] Piperno D.R., Ranere A.J., Holst I., Iriarte J., Dickau R. (2009). Starch grain and phytolith evidence for early ninth millennium B.P. Maize from the Central Balsas River Valley, Mexico. Proc. Natl. Acad. Sci. USA.

[12] Doebley J., Stec A. (1991). Genetic analysis of the morphological differences between maize and teosinte. Genetics.

[13] Doebley J., Stec A., Wendel J., Edwards M. (1990). Genetic and morphological analysis of a maize-teosinte F_2_ population: Implications for the origin of maize. Proc. Natl. Acad. Sci. USA.

[14] Doebley J.F. (1983). The maize and teosinte male inflorescence: A numerical taxonomic study. Ann. Mo. Bot. Gard..

[15] Guei R.G., Wassom C.E. (1996). Genetic analysis of tassel size and leaf senescence and their relationships with yield in two tropical lowland maize populations. Afr. Crop Sci. J..

[16] Brewbaker J.L. (2015). Diversity and genetics of tassel branch numbers in maize. Crop Sci..

[17] Duvick D.N., Cassman K.G. (1999). Post–green revolution trends in yield potential of temperate maize in the north-central united states. Crop Sci..

[18] Wang B., Lin Z., Li X., Zhao Y., Zhao B., Wu G., Ma X., Wang H., Xie Y., Li Q. (2020). Genome-wide selection and genetic improvement during modern maize breeding. Nat. Genet..

[19] Gao S.B., Zhao M.J., Hai L., Zhang Z.M. (2007). Identification of QTL associated with tassel branch number and total tassel length in maize. Hereditas.

[20] Mickelson S.M., Stuber C.S., Senior L., Kaeppler S.M. (2002). Quantitative trait loci controlling leaf and tassel traits in a B73 x Mo17 population of maize. Crop Sci..

[21] Upadyayula N., Wassom J., Bohn M.O., Rocheford T.R. (2006). Quantitative trait loci analysis of phenotypic traits and principal components of maize tassel inflorescence architecture. Theor. Appl. Genet..

[22] Li Y., Dong Y., Niu S., Cui D., Wang Y., Liu Y., Wei M., Li X. (2008). Identification of agronomically favorable quantitative trait loci alleles from a dent corn inbred Dan232 using advanced backcross QTL analysis and comparison with the F_2:3_ population in popcorn. Mol. Breed..

[23] Nikoli A., Anelkovi V., Dodig D., Mici-Ignjatovi D. (2011). Quantitative trait loci for yield and morphological traits in maize under drought stress. Genetika.

[24] Chen Z., Wang B., Dong X., Liu H., Ren L., Chen J., Hauck A., Song W., Lai J. (2014). An ultra-high density bin-map for rapid QTL mapping for tassel and ear architecture in a large F_2_ maize population. BMC Genom..

[25] Rice B.R., Fernandes S.B., Lipka A.E. (2020). Multi-trait genome-wide association studies reveal loci associated with maize inflorescence and leaf architecture. Plant Cell Physiol..

[26] Xie Y., Wang X., Ren X., Yang X., Zhao R. (2019). A SNP-based high-density genetic map reveals reproducible QTLs for tassel-related traits in maize (*zea mays l*.). Trop. Plant Biol..

[27] Wang Y., Chen J., Guan Z., Zhang X., Zhang Y., Ma L., Yao Y., Peng H., Zhang Q., Zhang B. (2019). Combination of multi-locus genome-wide association study and QTL mapping reveals genetic basis of tassel architecture in maize. Mol. Genet. Genom..

[28] Wu X., Li Y.X., Shi Y.S., Song Y.C., Zhang D.F., Li C.H., Buckler E.S., Li Y., Zhang Z.W., Wang T.Y. (2016). Joint-linkage mapping and GWAS reveal extensive genetic loci that regulate male inflorescence size in maize. Plant Biotechnol. J..

[29] Yang N., Lu Y.L., Yang X.H., Huang J., Zhou Y., Ali F., Wen W.W., Liu J., Li J.S., Yan J.B. (2014). Genome wide association studies using a new nonparametric model reveal the genetic architecture of 17 agronomic traits in an enlarged maize association panel. PLoS Genet..

[30] Gage J.L., White M.R., Edwards J.W., Kaeppler S., de Leon N. (2018). Selection signatures underlying dramatic male inflorescence transformation during modern hybrid maize breeding. Genetics.

[31] Pan Q.C., Xu Y.C., Li K., Peng Y., Zhan W., Li W.Q., Li L., Yan J.B. (2017). The genetic basis of plant architecture in 10 maize recombinant inbred line populations. Plant Physiol..

[32] Yi Q., Liu Y., Zhang X., Hou X., Zhang J., Liu H., Hu Y., Yu G., Huang Y. (2018). Comparative mapping of quantitative trait loci for tassel-related traits of maize in F_2:3_ and ril populations. J. Genet..

[33] Brown P.J., Upadyayula N., Mahone G.S., Tian F., Bradbury P.J., Myles S., Holland J.B., Flint-Garcia S., McMullen M.D., Buckler E.S. (2011). Distinct genetic architectures for male and female inflorescence traits of maize. PLoS Genet..

[34] Liu X., Hao L., Kou S., Su E., Zhou Y., Wang R., Mohamed A., Gao C., Zhang D., Li Y. (2018). High-density quantitative trait locus mapping revealed genetic architecture of leaf angle and tassel size in maize. Mol. Breed..

[35] Bouchet S., Bertin P., Presterl T., Jamin P., Coubriche D., Gouesnard B., Laborde J., Charcosset A. (2017). Association mapping for phenology and plant architecture in maize shows higher power for developmental traits compared with growth influenced traits. Heredity.

[36] Liu Y., Hou X., Xiao Q., Yi Q., Bian S., Hu Y., Liu H., Zhang J., Hao X., Cheng W. (2016). Genetic analysis in maize foundation parents with mapping population and testcross population: Ye478 carried more favorable alleles and using QTL information could improve foundation parents. Front. Plant Sci..

[37] Zhao X., Peng Y., Zhang J., Fang P., Wu B. (2017). Mapping QTLs and meta-QTLs for two inflorescence architecture traits in multiple maize populations under different watering environments. Mol. Breed..

[38] Wilson Z.A., Zhang D.B. (2009). From *Arabidopsis* to rice: Pathways in pollen development. J. Exp. Bot..

[39] Yuan L. (1994). Purification and production of foundation seed of rice PGMS and TGMS lines. Rice.

[40] Cheng S.H., Zhuang J.Y., Fan Y.Y., Du J.H., Cao L.Y. (2007). Progress in research and development on hybrid rice: A super-domesticate in china. Ann. Bot..

[41] Wise R.P., Gobelman-Werner K., Pei D., Dill C.L., Schnable P.S. (1999). Mitochondrial transcript processing and restoration of male fertility in T-cytoplasm maize. J. Hered..

[42] Wu Y., Fox T.W., Trimnell M.R., Wang L., Xu R., Cigan A.M., Huffman G.A., Garnaat C.W., Hershey H., Albertsen M.C. (2016). Development of a novel recessive genetic male sterility system for hybrid seed production in maize and other cross-pollinating crops. Plant Biotechnol. J..

[43] Wan X., Wu S., Li Z., Dong Z., Li J. (2019). Maize genic male-sterility genes and their applications in hybrid breeding: Progress and perspectives. Mol. Plant.

[44] Wan X., Wu S., Li X. (2021). Breeding with dominant genic male-sterility genes to boost crop grain yield in the post-heterosis utilization era. Mol. Plant.

[45] Ma H. (2005). Molecular genetic analyses of microsporogenesis and microgametogenesis in flowering plants. Annu. Rev. Plant Biol..

[46] Zhang D., Luo X., Zhu L. (2011). Cytological analysis and genetic control of rice anther development. J. Genet. Genom..

[47] van der Linde K., Walbot V. (2019). Pre-meiotic anther development. Curr. Top. Dev. Biol..

[48] Wan X., Wu S., Li Z., An X., Tian Y. (2020). Lipid metabolism: Critical roles in male fertility and other aspects of reproductive development in plants. Mol. Plant.

[49] Liu S., Li Z., Wu S., Wan X. (2021). The essential roles of sugar metabolism for pollen development and male fertility in plants. Crop J..

[50] Gómez J.F., Talle B., Wilson Z.A. (2015). Anther and pollen development: A conserved developmental pathway. J. Integr. Plant Biol..

[51] Chaudhury A.M. (1993). Nuclear genes controlling male fertility. Plant Cell.

[52] Ariizumi T., Toriyama K. (2011). Genetic regulation of sporopollenin synthesis and pollen exine development. Annu. Rev. Plant Biol..

[53] Li Z., An X., Zhu T., Yan T., Wu S., Tian Y., Li J., Wan X. (2019). Discovering and constructing cerna-mirna-target gene regulatory networks during anther development in maize. Int. J. Mol. Sci..

[54] Ma J., Skibbe D.S., Fernandes J., Walbot V. (2008). Male reproductive development: Gene expression profiling of maize anther and pollen ontogeny. Genome Biol..

[55] Nelms B., Walbot V. (2019). Defining the developmental program leading to meiosis in maize. Science.

[56] Nelms B., Walbot V. (2022). Gametophyte genome activation occurs at pollen mitosis i in maize. Science.

[57] Wang C., Yang X., Li G. (2021). Molecular insights into inflorescence meristem specification for yield potential in cereal crops. Int. J. Mol. Sci..

[58] Du Y., Wu B., Xing Y., Zhang Z. (2022). Conservation and divergence: Regulatory networks underlying reproductive branching in rice and maize. J. Adv. Res..

[59] Zhang D., Yuan Z. (2014). Molecular control of grass inflorescence development. Annu. Rev. Plant Biol..

[60] Li M., Zhong W., Yang F., Zhang Z. (2018). Genetic and molecular mechanisms of quantitative trait loci controlling maize inflorescence architecture. Plant Cell Physiol..

[61] Gaillochet C., Daum G., Lohmann J.U. (2015). O cell, where art thou? The mechanisms of shoot meristem patterning. Curr. Opin. Plant Biol..

[62] Tucker M.R., Laux T. (2007). Connecting the paths in plant stem cell regulation. Trends Cell Biol..

[63] Hake S. (2008). Inflorescence architecture: The transition from branches to flowers. Curr. Biol..

[64] Yao H., Skirpan A., Wardell B., Matthes M.S., Best N.B., McCubbin T., Durbak A., Smith T., Malcomber S., McSteen P. (2019). The *barren stalk2* gene is required for axillary meristem development in maize. Mol. Plant.

[65] Chongloi G.L., Prakash S., Vijayraghavan U. (2019). Regulation of meristem maintenance and organ identity during rice reproductive development. J. Exp. Bot..

[66] Chang M.T., Neuffer M.G. (1994). Chromosomal Behavior during Microsporogenesis.

[67] Huang A. (2011). Transcriptomes of the anther sporophyte: Availability and uses. Plant Cell Physiol..

[68] Jiang Y., An X., Li Z., Yan T., Zhu T., Xie K., Liu S., Hou Q., Zhao L., Wu S. (2021). CRISPR/Cas9-based discovery of maize transcription factors regulating male sterility and their functional conservation in plants. Plant Biotechnol. J..

[69] Zhou L.-Z., Juranić M., Dresselhaus T. (2017). Germline development and fertilization mechanisms in maize. Mol. Plant.

[70] Bommert P., Je B.I., Goldshmidt A., Jackson D. (2013). The maize Galpha gene *COMPACT PLANT2* functions in CLAVATA signalling to control shoot meristem size. Nature.

[71] Taguchi-Shiobara F., Yuan Z., Hake S., Jackson D. (2001). The *fasciated ear2* gene encodes a leucine-rich repeat receptor-like protein that regulates shoot meristem proliferation in maize. Genes Dev..

[72] Je B.I., Gruel J., Lee Y.K., Bommert P., Arevalo E.D., Eveland A.L., Wu Q., Goldshmidt A., Meeley R., Bartlett M. (2016). Signaling from maize organ primordia via FASCIATED EAR3 regulates stem cell proliferation and yield traits. Nat. Genet..

[73] Pautler M., Eveland A.L., LaRue T., Yang F., Weeks R., Lunde C., Il Je B., Meeley R., Komatsu M., Vollbrecht E. (2015). *FASCIATED EAR4* encodes a bZIP transcription factor that regulates shoot meristem size in maize. Plant Cell.

[74] Bommert P., Lunde C., Nardmann J., Vollbrecht E., Running M., Jackson D., Hake S., Werr W. (2005). *thick tassel dwarf1* encodes a putative maize ortholog of the *Arabidopsis CLAVATA1* leucine-rich repeat receptor-like kinase. Development.

[75] Xie S., Luo H., Huang Y., Wang Y., Ru W., Shi Y., Huang W., Wang H., Dong Z., Jin W. (2020). A missense mutation in a large subunit of ribonucleotide reductase confers temperature-gated tassel formation. Plant Physiol..

[76] Wu Q., Xu F., Liu L., Char S.N., Ding Y., Je B.I., Schmelz E., Yang B., Jackson D. (2020). The maize heterotrimeric G protein β subunit controls shoot meristem development and immune responses. Proc. Natl. Acad. Sci. USA.

[77] Nardmann J., Werr W. (2006). The shoot stem cell niche in angiosperms: Expression patterns of *WUS* orthologues in rice and maize imply major modifications in the course of mono- and dicot evolution. Mol. Biol. Evol..

[78] Ritter M.K., Padilla C.M., Schmidt R.J. (2002). The maize mutant *barren stalk1* is defective in axillary meristem development. Am. J. Bot..

[79] Gallavotti A., Zhao Q., Kyozuka J., Meeley R.B., Ritter M., Doebley J.F., Pe M.E., Schmidt R.J. (2004). The role of *barren stalk1* in the architecture of maize. Nature.

[80] Barazesh S., McSteen P. (2008). *Barren inflorescence1* functions in organogenesis during vegetative and inflorescence development in maize. Genetics.

[81] Galli M., Liu Q., Moss B.L., Malcomber S., Li W., Gaines C., Federici S., Roshkovan J., Meeley R., Nemhauser J.L. (2015). Auxin signaling modules regulate maize inflorescence architecture. Proc. Natl. Acad. Sci. USA.

[82] McSteen P., Hake S. (2001). *barren inflorescence2* regulates axillary meristem development in the maize inflorescence. Development.

[83] McSteen P., Malcomber S., Skirpan A., Lunde C., Wu X.T., Kellogg E., Hake S. (2007). *barren inflorescence2* encodes a co-ortholog of the *PINOID* serine/threonine kinase and is required for organogenesis during inflorescence and vegetative development in maize. Plant Physiol..

[84] Skirpan A., Wu X.T., McSteen P. (2008). Genetic and physical interaction suggest that BARREN STALK1 is a target of BARREN INFLORESCENCE2 in maize inflorescence development. Plant J..

[85] Vollbrecht E., Reiser L., Hake S. (2000). Shoot meristem size is dependent on inbred background and presence of the maize homeobox gene, knotted1. Development.

[86] Bolduc N., Yilmaz A., Mejia-Guerra M.K., Morohashi K., O’Connor D., Grotewold E., Hake S. (2012). Unraveling the KNOTTED1 regulatory network in maize meristems. Genes Dev..

[87] Bolduc N., Hake S. (2009). The maize transcription factor KNOTTED1 directly regulates the gibberellin catabolism gene *ga2ox1*. Plant Cell.

[88] Walsh J., Freeling M. (1999). The liguleless2 gene of maize functions during the transition from the vegetative to the reproductive shoot apex. Plant J..

[89] Qin X., Tian S., Zhang W., Dong X., Ma C., Wang Y., Yan J., Yue B. (2020). *Q^dtbn1^*, an F-box gene affecting maize tassel branch number by a dominant model. Plant Biotechnol. J..

[90] Chatterjee M., Tabi Z., Galli M., Malcomber S., Buck A., Muszynski M., Gallavotti A. (2014). The boron efflux transporter ROTTEN EAR is required for maize inflorescence development and fertility. Plant Cell.

[91] Gallavotti A., Barazesh S., Malcomber S., Hall D., Jackson D., Schmidt R.J., McSteen P. (2008). *sparse inflorescence1* encodes a monocot-specific *YUCCA*-like gene required for vegetative and reproductive development in maize. Proc. Natl. Acad. Sci. USA.

[92] Leonard A., Holloway B., Guo M., Rupe M., Yu G.X., Beatty M., Zastrow-Hayes G., Meeley R., Llaca V., Butler K. (2014). *tassel-less1* encodes a boron channel protein required for inflorescence development in maize. Plant Cell Physiol..

[93] Chuck G., Whipple C., Jackson D., Hake S. (2010). The maize SBP-box transcription factor encoded by *tasselsheath4* regulates bract development and the establishment of meristem boundaries. Development.

[94] Han J.J., Jackson D., Martienssen R. (2012). Pod corn is caused by rearrangement at the *Tunicate1* locus. Plant Cell.

[95] Chuck G.S., Brown P.J., Meeley R., Hake S. (2014). Maize *SBP-box* transcription factors *unbranched2* and *unbranched3* affect yield traits by regulating the rate of lateral primordia initiation. Proc. Natl. Acad. Sci. USA.

[96] Du Y., Liu L., Li M., Fang S., Shen X., Chu J., Zhang Z. (2017). *UNBRANCHED3* regulates branching by modulating cytokinin biosynthesis and signaling in maize and rice. New Phytol..

[97] Phillips K.A., Skirpan A.L., Liu X., Christensen A., Slewinski T.L., Hudson C., Barazesh S., Cohen J.D., Malcomber S., McSteen P. (2011). *vanishing tassel2* encodes a grass-specific tryptophan aminotransferase required for vegetative and reproductive development in maize. Plant Cell.

[98] Bomblies K., Wang R.L., Ambrose B.A., Schmidt R.J., Meeley R.B., Doebley J. (2003). Duplicate *FLORICAULA/LEAFY* homologs *zfl1* and *zfl2* control inflorescence architecture and flower patterning in maize. Development.

[99] Chuck G., Muszynski M., Kellogg E., Hake S., Schmidt R.J. (2002). The control of spikelet meristem identity by the *branched silkless1* gene in maize. Science.

[100] Kaplinsky N.J. (2003). Combinatorial control of meristem identity in maize inflorescences. Development.

[101] Chuck G., Meeley R., Hake S. (2008). Floral meristem initiation and meristem cell fate are regulated by the maize *AP2* genes *ids1* and *sid1*. Development.

[102] Hartwig T., Chuck G.S., Fujioka S., Klempien A., Weizbauer R., Potluri D.P.V., Choe S., Johal G.S., Schulz B. (2011). Brassinosteroid control of sex determination in maize. Proc. Natl. Acad. Sci. USA.

[103] Vollbrecht E., Springer P.S., Goh L., Buckler E.S., Martienssen R. (2005). Architecture of floral branch systems in maize and related grasses. Nature.

[104] Bortiri E., Chuck G., Vollbrecht E., Rocheford T., Martienssen R., Hake S. (2006). *ramosa2* encodes a LATERAL ORGAN BOUNDARY domain protein that determines the fate of stem cells in branch meristems of maize. Plant Cell.

[105] Satoh-Nagasawa N., Nagasawa N., Malcomber S., Sakai H., Jackson D. (2006). A trehalose metabolic enzyme controls inflorescence architecture in maize. Nature.

[106] Gallavotti A., Long J.A., Stanfield S., Yang X., Jackson D., Vollbrecht E., Schmidt R.J. (2010). The control of axillary meristem fate in the maize *ramosa* pathway. Development.

[107] Luo H., Meng D., Liu H., Xie M., Yin C., Liu F., Dong Z., Jin W. (2020). Ectopic expression of the transcriptional regulator *silky3* causes pleiotropic meristem and sex determination defects in maize inflorescences. Plant Cell.

[108] DeLong A., Calderon-Urrea A., Dellaporta S.L. (1993). Sex determination gene *TASSELSEED2* of maize encodes a short-chain alcohol dehydrogenase required for stage-specific floral organ abortion. Cell.

[109] Chuck G., Cigan A.M., Saeteurn K., Hake S. (2007). The heterochronic maize mutant *Corngrass1* results from overexpression of a tandem microRNA. Nat. Genet..

[110] Thompson B.E., Basham C., Hammond R., Ding Q.Y., Kakrana A., Lee T.F., Simon S.A., Meeley R., Meyers B.C., Hake S. (2014). The *dicer-like1* Homolog *fuzzy tassel* is required for the regulation of meristem determinacy in the inflorescence and vegetative growth in maize. Plant Cell.

[111] Chuck G., Meeley R., Irish E., Sakai H., Hake S. (2007). The maize *tasselseed4* microRNA controls sex determination and meristem cell fate by targeting *Tasselseed6*/*indeterminate spikelet1*. Nat. Genet..

[112] Bai F., Reinheimer R., Durantini D., Kellogg E.A., Schmidt R.J. (2012). TCP transcription factor, BRANCH ANGLE DEFECTIVE 1 (BAD1), is required for normal tassel branch angle formation in maize. Proc. Natl. Acad. Sci. USA.

[113] Gallavotti A., Malcomber S., Gaines C., Stanfield S., Whipple C., Kellogg E., Schmidt R.J. (2011). BARREN STALK FASTIGIATE1 is an AT-Hook protein required for the formation of maize ears. Plant Cell.

[114] Lewis M.W., Bolduc N., Hake K., Htike Y., Hay A., Candela H., Hake S. (2014). Gene regulatory interactions at lateral organ boundaries in maize. Development.

[115] Whipple C.J., Hall D.H., DeBlasio S., Taguchi-Shiobara F., Schmidt R.J., Jackson D.P. (2010). A conserved mechanism of bract suppression in the grass family. Plant Cell.

[116] Schoof H., Lenhard M., Haecker A., Mayer K.F.X., Jürgens G., Laux T. (2000). The stem cell population of *Arabidopsis* shoot meristems is maintained by a regulatory loop between the *CLAVATA* and *WUSCHEL* genes. Cell.

[117] Galli M., Gallavotti A. (2016). Expanding the regulatory network for meristem size in plants. Trends Genet..

[118] Brand U., Grunewald M., Hobe M., Simon R. (2002). Regulation of *CLV3* expression by two homeobox genes in *Arabidopsis*. Plant Physiol..

[119] Trotochaud A.E., Jeong S., Clark S.E. (2000). CLAVATA3, a multimeric ligand for the CLAVATA1 receptor-kinase. Science.

[120] Fletcher J.C., Brand U., Running M.P., Simon R., Meyerowitz E.M. (1999). Signaling of cell fate decisions by *CLAVATA3* in *Arabidopsis* shoot meristems. Science.

[121] Hirakawa Y. (2021). CLAVATA3, a plant peptide controlling stem cell fate in the meristem. Peptides.

[122] Brand U., Fletcher J.C., Hobe M., Meyerowitz E.M., Simon R. (2000). Dependence of stem cell fate in *Arabidopsis* on a feedback loop regulated by *CLV3* activity. Science.

[123] Nimchuk Z.L., Zhou Y., Tarr P.T., Peterson B.A., Meyerowitz E.M. (2015). Plant stem cell maintenance by transcriptional cross-regulation of related receptor kinases. Development.

[124] Hu C., Zhu Y., Cui Y., Cheng K., Liang W., Wei Z., Zhu M., Yin H., Zeng L., Xiao Y. (2018). A group of receptor kinases are essential for CLAVATA signalling to maintain stem cell homeostasis. Nat. Plants.

[125] Muller R., Bleckmann A., Simon R. (2008). The receptor kinase CORYNE of *Arabidopsis* transmits the stem cell-limiting signal CLAVATA3 independently of CLAVATA1. Plant Cell.

[126] Chen Z., Li W., Gaines C., Buck A., Galli M., Gallavotti A. (2021). Structural variation at the maize *WUSCHEL1* locus alters stem cell organization in inflorescences. Nat. Commun..

[127] Bommert P., Nagasawa N.S., Jackson D. (2013). Quantitative variation in maize kernel row number is controlled by the *FASCIATED EAR2* locus. Nat. Genet..

[128] Je B.I., Xu F., Wu Q., Liu L., Meeley R., Gallagher J.P., Corcilius L., Payne R.J., Bartlett M.E., Jackson D. (2018). The CLAVATA receptor FASCIATED EAR2 responds to distinct CLE peptides by signaling through two downstream effectors. eLife.

[129] Tsuda K., Abraham-Juarez M.J., Maeno A., Dong Z., Aromdee D., Meeley R., Shiroishi T., Nonomura K.I., Hake S. (2017). KNOTTED1 cofactors, BLH12 and BLH14, regulate internode patterning and vein anastomosis in maize. Plant Cell.

[130] Yang R.S., Xu F., Wang Y.M., Zhong W.S., Dong L., Shi Y.N., Tang T.J., Sheng H.J., Jackson D., Yang F. (2021). Glutaredoxins regulate maize inflorescence meristem development via redox control of TGA transcriptional activity. Nat. Plants.

[131] Yang F., Bui H.T., Pautler M., Llaca V., Johnston R., Lee B.H., Kolbe A., Sakai H., Jackson D. (2015). A maize glutaredoxin gene, *abphyl2*, regulates shoot meristem size and phyllotaxy. Plant Cell.

[132] Zeng J., Dong Z., Wu H., Tian Z., Zhao Z. (2017). Redox regulation of plant stem cell fate. EMBO J..

[133] Salehin M., Bagchi R., Estelle M. (2015). SCF^TIR1/AFB^-based auxin perception: Mechanism and role in plant growth and development. Plant Cell.

[134] Skirpan A., Culler A.H., Gallavotti A., Jackson D., Cohen J.D., McSteen P. (2009). BARREN INFLORESCENCE2 interaction with ZmPIN1a suggests a role in auxin transport during maize inflorescence development. Plant Cell Physiol..

[135] Friml J., Yang X., Michniewicz M., Weijers D., Quint A., Tietz O. (2004). A PINOID-dependent binary switch in apical-basal PIN polar targeting directs auxin efflux. Science.

[136] Tabuchi H., Zhang Y., Hattori S., Omae M., Shimizu-Sato S., Oikawa T., Qian Q., Nishimura M., Kitano H., Xie H. (2011). *LAX PANICLE2* of rice encodes a novel nuclear protein and regulates the formation of axillary meristems. Plant Cell.

[137] Walsh J., Waters C.A., Freeling M. (1998). The maize gene *liguleless2* encodes a basic leucine zipper protein involved in the establishment of the leaf blade-sheath boundary. Genes Dev..

[138] Mcsteen P. (2006). Branching out: The ramosa pathway and the evolution of grass inflorescence morphology. Plant Cell.

[139] Kellogg E.A. (2007). Floral displays: Genetic control of grass inflorescences. Curr. Opin. Plant Biol..

[140] Wu X., Skirpan A., McSteen P. (2009). *Suppressor of sessile spikelets1* functions in the ramosa pathway controlling meristem determinacy in maize. Plant Physiol..

[141] Krol J., Loedige I., Filipowicz W. (2010). The widespread regulation of microRNA biogenesis, function and decay. Nat. Rev. Genet..

[142] Schauer S.E., Jacobsen S.E., Meinke D.W., Ray A. (2002). *DICER-LIKE1*: Blind men and elephants in *Arabidopsis* development. Trends Plant Sci..

[143] Liu B., Li P., Li X., Liu C., Cao S., Chu C., Cao X. (2005). Loss of function of *OsDCL1* affects microRNA accumulation and causes developmental defects in rice. Plant Physiol..

[144] Moreno M.A., Harper L.C., Krueger R.W., Dellaporta S.L., Freeling M. (1997). Liguleless1 encodes a nuclear-localized protein required for induction of ligules and auricles during maize leaf organogenesis. Genes Dev..

[145] Xiao Y.J., Liu H.J., Wu L.J., Warburton M., Yan J.B. (2017). Genome-wide association studies in maize: Praise and stargaze. Mol. Plant.

[146] Maher B. (2008). Personal genomes: The case of the missing heritability. Nature.

[147] Eichler E.E., Flint J., Gibson G., Kong A., Leal S.M., Moore J.H., Nadeau J.H. (2010). Missing heritability and strategies for finding the underlying causes of complex disease. Nat. Rev. Genet..

[148] Liu L., Gallagher J., Arevalo E.D., Chen R., Skopelitis T., Wu Q., Bartlett M., Jackson D. (2021). Enhancing grain-yield-related traits by CRISPR-Cas9 promoter editing of maize *CLE* genes. Nat. Plants.

[149] Timofejeva L., Skibbe D., Lee S., Golubovskaya I., Wang C.-J., Harper L., Walbot V., Cande W. (2013). Cytological characterization and allelism testing of anther developmental mutants identified in a screen of maize male sterile lines. G3-Genes Genom Genet..

[150] Liu X., Zhang S., Jiang Y., Yan T., Fang C., Hou Q., Wu S., Xie K., An X., Wan X. (2022). Use of CRISPR/Cas9-based gene editing to simultaneously mutate multiple homologous genes required for pollen development and male fertility in maize. Cells.

[151] Nan G.-L., Teng C., Fernandes J., O’Connor L., Meyers B.C., Walbot V. (2022). A cascade of bHLH-regulated pathways programs maize anther development. Plant Cell.

[152] Vernoud V., Laigle G., Rozier F., Meeley R.B., Perez P., Rogowsky P.M. (2009). The HD-ZIP IV transcription factor OCL4 is necessary for trichome patterning and anther development in maize. Plant J..

[153] Murmu J., Bush M.J., Delong C., Li S., Xu M., Khan M., Malcolmson C., Fobert P.R., Zachgo S., Hepworth S.R. (2010). *Arabidopsis* basic leucine-zipper transcription factors TGA9 and TGA10 interact with floral glutaredoxins *ROXY1* and *ROXY2* and are redundantly required for anther development. Plant Physiol..

[154] Chen Z.S., Liu X.F., Wang D.H., Chen R., Zhang X., Xu Z.H., Bai S.N. (2017). Transcription factor OsTGA10 is a target of the MADS protein OsMADS8 and is required for tapetum development. Plant Physiol..

[155] Moon J., Skibbe D., Timofejeva L., Wang C.J., Kelliher T., Kremling K., Walbot V., Cande W.Z. (2013). Regulation of cell divisions and differentiation by MALE STERILITY32 is required for anther development in maize. Plant J..

[156] Jung K.H., Han M.J., Lee Y.S., Kim Y.W., An G. (2005). Rice *Undeveloped Tapetum1* is a major regulator of early tapetum development. Plant Cell.

[157] Wei Z., Sun Y., Timofejeva L., Chen C., Ma H. (2006). Regulation of *Arabidopsis* tapetum development and function by *DYSFUNCTIONAL TAPETUM1 (DYT1)* encoding a putative bHLH transcription factor. Development.

[158] Na L., Zhang D.S., Liu H.S., Yin C.S., Zhang D.B. (2006). The rice *tapetum degeneration retardation* gene is required for tapetum degradation and anther development. Plant Cell.

[159] Nan G.L., Zhai J., Arikit S., Morrow D., Fernandes J., Mai L., Nguyen N., Meyers B.C., Walbot V. (2017). MS23, a master basic helix-loop-helix factor, regulates the specification and development of the tapetum in maize. Development.

[160] Fu Z., Yu J., Cheng X., Zong X., Xu J., Chen M., Li Z., Zhang D., Liang W. (2014). The rice basic helix-loop-helix transcription factor TDR interacting protein2 is a central switch in early anther development. Plant Cell.

[161] Ko S.S., Li M.J., Ku S.B., Ho Y.C., Lin Y.J., Chuang M.H., Hsing H.X., Lien Y.C., Yang H.T., Chang H.C. (2014). The bHLH142 transcription factor coordinates with TDR1 to modulate the expression of *EAT1* and regulate pollen development in rice. Plant Cell.

[162] Zhu J., Chen H., Li H., Gao J.F., Jiang H., Wang C., Guan Y.F., Yang Z.N. (2008). *Defective in tapetal development and function 1* is essential for anther development and tapetal function for microspore maturation in *Arabidopsis*. Plant J..

[163] Cai C.-F., Zhu J., Lou Y., Guo Z.-L., Xiong S.-X., Wang K., Yang Z.-N. (2015). The functional analysis of *OsTDF1* reveals a conserved genetic pathway for tapetal development between rice and *Arabidopsis*. Sci. Bull..

[164] Millar A.A., Gubler F. (2005). The *Arabidopsis GAMYB-like* genes, *MYB33* and *MYB65*, are microRNA-regulated genes that redundantly facilitate anther development. Plant Cell.

[165] Phan H.A., Li S.F., Parish R.W. (2012). MYB80, a regulator of tapetal and pollen development, is functionally conserved in crops. Plant Mol. Biol..

[166] Pan X., Yan W., Chang Z., Xu Y., Luo M., Xu C., Chen Z., Wu J., Tang X. (2020). OsMYB80 regulates anther development and pollen fertility by targeting multiple biological pathways. Plant Cell Physiol..

[167] Yang X., Makaroff C.A., Ma H. (2003). The *Arabidopsis MALE MEIOCYTE DEATH1* gene encodes a PHD-finger protein that is required for male meiosis. Plant Cell.

[168] Yang Z., Sun L., Zhang P., Zhang Y., Yu P., Liu L., Abbas A., Xiang X., Wu W., Zhan X. (2019). *TDR interacting protein 3*, encoding a PHD-finger transcription factor, regulates ubisch bodies and pollen wall formation in rice. Plant J..

[169] Zhang D., Wu S., An X., Xie K., Dong Z., Zhou Y., Xu L., Fang W., Liu S., Liu S. (2018). Construction of a multicontrol sterility system for a maize male-sterile line and hybrid seed production based on the *ZmMs7* gene encoding a PHD-finger transcription factor. Plant Biotechnol. J..

[170] An X., Ma B., Duan M., Dong Z., Liu R., Yuan D., Hou Q., Wu S., Zhang D., Liu D. (2020). Molecular regulation of *ZmMs7* required for maize male fertility and development of a dominant male-sterility system in multiple species. Proc. Natl. Acad. Sci. USA.

[171] Evans M.M. (2007). The *indeterminate gametophyte1* gene of maize encodes a LOB domain protein required for embryo sac and leaf development. Plant Cell.

[172] Kim M.J., Kim M., Lee M.R., Park S.K., Kim J. (2015). *LATERAL ORGAN BOUNDARIES DOMAIN* (*LBD*)*10* interacts with *SIDECAR POLLEN*/*LBD27* to control pollen development in *Arabidopsis*. Plant J..

[173] Xie K., Wu S., Li Z., Zhou Y., Zhang D., Dong Z., An X., Zhu T., Zhang S., Liu S. (2018). Map-based cloning and characterization of *zea mays male sterility33* (*ZmMs33*) gene, encoding a glycerol-3-phosphate acyltransferase. Theor. Appl. Genet..

[174] Zhu T., Wu S., Zhang D., Li Z., Xie K., An X., Ma B., Hou Q., Dong Z., Tian Y. (2019). Genome-wide analysis of maize *GPAT* gene family and cytological characterization and breeding application of *ZmMs33*/*ZmGPAT6* gene. Theor. Appl. Genet..

[175] Zhu T., Li Z., An X., Long Y., Xue X., Xie K., Ma B., Zhang D., Guan Y., Niu C. (2020). Normal structure and function of endothecium chloroplasts maintained by ZmMs33-mediated lipid biosynthesis in tapetal cells are critical for anther development in maize. Mol. Plant.

[176] Djukanovic V., Smith J., Lowe K., Yang M., Gao H., Jones S., Nicholson M.G., West A., Lape J., Bidney D. (2013). Male-sterile maize plants produced by targeted mutagenesis of the cytochrome p450-like gene (*MS26*) using a re-designed i-crei homing endonuclease. Plant J..

[177] Somaratne Y., Tian Y., Zhang H., Wang M., Huo Y., Cao F., Zhao L., Chen H. (2017). ABNORMAL POLLEN VACUOLATION1 (APV1) is required for male fertility by contributing to anther cuticle and pollen exine formation in maize. Plant J..

[178] An X., Dong Z., Tian Y., Xie K., Wu S., Zhu T., Zhang D., Zhou Y., Niu C., Ma B. (2019). *ZmMs30* encoding a novel GDSL lipase is essential for male fertility and valuable for hybrid breeding in maize. Mol. Plant.

[179] Fox T., DeBruin J., Haug Collet K., Trimnell M., Clapp J., Leonard A., Li B., Scolaro E., Collinson S., Glassman K. (2017). A single point mutation in *Ms44* results in dominant male sterility and improves nitrogen use efficiency in maize. Plant Biotechnol. J..

[180] Chen X., Zhang H., Sun H., Luo H., Zhao L., Dong Z., Yan S., Zhao C., Liu R., Xu C. (2017). IRREGULAR POLLEN EXINE1 is a novel factor in anther cuticle and pollen exine formation. Plant Physiol..

[181] Wang Y., Liu D., Tian Y., Wu S., An X., Dong Z., Zhang S., Bao J., Li Z., Li J. (2019). Map-based cloning, phylogenetic, and microsynteny analyses of *ZmMs20* gene regulating male fertility in maize. Int. J. Mol. Sci..

[182] Liu Z., Lin S., Shi J., Yu J., Zhu L., Yang X., Zhang D., Liang W. (2017). *Rice no pollen 1* (*NP1*) is required for anther cuticle formation and pollen exine patterning. Plant J..

[183] Huo Y., Pei Y., Tian Y., Zhang Z., Li K., Liu J., Xiao S., Chen H., Liu J. (2020). *IRREGULAR POLLEN EXINE2* encodes a GDSL lipase essential for male fertility in maize. Plant Physiol..

[184] Tian Y., Xiao S., Liu J., Somaratne Y., Zhang H., Wang M., Zhang H., Zhao L., Chen H. (2017). *MALE STERILE6021* (*MS6021*) is required for the development of anther cuticle and pollen exine in maize. Sci. Rep..

[185] Jiang Y., Li Z., Liu X., Zhu T., Xie K., Hou Q., Yan T., Niu C., Zhang S., Yang M. (2021). *ZmFAR1* and *ZmABCG26* regulated by microRNA are essential for lipid metabolism in maize anther. Int. J. Mol. Sci..

[186] Zhang S., Wu S., Niu C., Liu D., Yan T., Tian Y., Liu S., Xie K., Li Z., Wang Y. (2021). *ZmMs25* encoding a plastid-localized fatty acyl reductase is critical for anther and pollen development in maize. J. Exp. Bot..

[187] Cigan A.M., Unger E., Xu R.J., Kendall T., Fox T.W. (2001). Phenotypic complementation of *ms45* maize requires tapetal expression of MS45. Sex. Plant Reprod..

[188] Xu Q., Yang L., Kang D., Ren Z., Liu Y. (2021). Maize *MS2* encodes an ATP-binding cassette transporter that is essential for anther development. Crop J..

[189] de Azevedo Souza C., Kim S.S., Koch S., Kienow L., Schneider K., McKim S.M., Haughn G.W., Kombrink E., Douglas C.J. (2009). A novel fatty acyl-CoA synthetase is required for pollen development and sporopollenin biosynthesis in *Arabidopsis*. Plant Cell.

[190] Yang X., Liang W., Chen M., Zhang D., Zhao X., Shi J. (2017). Rice fatty acyl-CoA synthetase OsACOS12 is required for tapetum programmed cell death and male fertility. Planta.

[191] Li Y., Li D., Guo Z., Shi Q., Xiong S., Zhang C., Zhu J., Yang Z. (2016). *OsACOS12*, an orthologue of *Arabidopsis* acyl-CoA synthetase5, plays an important role in pollen exine formation and anther development in rice. BMC Plant Biol..

[192] Grienenberger E., Kim S.S., Lallemand B., Geoffroy P., Heintz D., Souza Cde A., Heitz T., Douglas C.J., Legrand M. (2010). Analysis of *TETRAKETIDE α-PYRONE REDUCTASE* function in *Arabidopsis thaliana* reveals a previously unknown, but conserved, biochemical pathway in sporopollenin monomer biosynthesis. Plant Cell.

[193] Xu D., Qu S., Tucker M.R., Zhang D., Liang W., Shi J. (2019). *Ostkpr1* functions in anther cuticle development and pollen wall formation in rice. BMC Plant Biol..

[194] Wang D., Oses-Prieto J.A., Li K.H., Fernandes J.F., Burlingame A.L., Walbot V. (2010). The *male sterile 8* mutation of maize disrupts the temporal progression of the transcriptome and results in the mis-regulation of metabolic functions. Plant J..

[195] Wang D., Skibbe D.S., Walbot V. (2013). Maize *Male sterile 8* (*Ms8*), a putative beta-1,3-galactosyltransferase, modulates cell division, expansion, and differentiation during early maize anther development. Plant Reprod.

[196] Suzuki T., Narciso J.O., Zeng W., van de Meene A., Yasutomi M., Takemura S., Lampugnani E.R., Doblin M.S., Bacic A., Ishiguro S. (2016). Kns4/upex1: A type II arabinogalactan *β*-(1,3)-galactosyltransferase required for pollen exine development. Plant Physiol..

[197] Chaubal R., Anderson J.R., Trimnell M.R., Fox T.W., Albertsen M.C., Bedinger P. (2003). The transformation of anthers in the *msca1* mutant of maize. Planta.

[198] Hong L., Tang D., Shen Y., Hu Q., Wang K., Li M., Lu T., Cheng Z. (2012). MIL2 (MICROSPORELESS2) regulates early cell differentiation in the rice anther. New Phytol..

[199] Xing S., Zachgo S. (2010). *ROXY1* and *ROXY2*, two *Arabidopsis* glutaredoxin genes, are required for anther development. Plant J..

[200] Wang C.J., Nan G.L., Kelliher T., Timofejeva L., Vernoud V., Golubovskaya I.N., Harper L., Egger R., Walbot V., Cande W.Z. (2012). Maize *multiple archesporial cells 1* (*mac1*), an ortholog of rice *TDL1A*, modulates cell proliferation and identity in early anther development. Development.

[201] Kelliher T., Walbot V. (2012). Hypoxia triggers meiotic fate acquisition in maize. Science.

[202] Hong L., Tang D., Zhu K., Wang K., Li M., Cheng Z. (2012). Somatic and reproductive cell development in rice anther is regulated by a putative glutaredoxin. Plant Cell.

[203] Chaubal R., Zanella C., Trimnell M.R., Fox T.W., Albertsen M.C., Bedinger P. (2000). Two male-sterile mutants of *zea mays* (Poaceae) with an extra cell division in the anther wall. Am. J. Bot..

[204] Lou Y., Zhou H.S., Han Y., Zeng Q.Y., Zhu J., Yang Z.N. (2018). Positive regulation of *AMS* by TDF1 and the formation of a TDF1-AMS complex are required for anther development in *Arabidopsis thaliana*. New Phytol..

[205] Gu J.N., Zhu J., Yu Y., Teng X.D., Lou Y., Xu X.F., Liu J.L., Yang Z.N. (2014). DYT1 directly regulates the expression of *TDF1* for tapetum development and pollen wall formation in *Arabidopsis*. Plant J..

[206] Zhu E., You C., Wang S., Cui J., Niu B., Wang Y., Qi J., Ma H., Chang F. (2015). The DYT1-interacting proteins bHLH010, bHLH089 and bHLH091 are redundantly required for *Arabidopsis* anther development and transcriptome. Plant J..

[207] Cui J., You C., Zhu E., Huang Q., Ma H., Chang F. (2016). Feedback regulation of DYT1 by interactions with downstream bHLH factors promotes DYT1 nuclear localization and anther development. Plant Cell.

[208] Zhang Z.B., Zhu J., Gao J.F., Wang C., Li H., Li H., Zhang H.Q., Zhang S., Wang D.M., Wang Q.X. (2007). Transcription factor *AtMYB103* is required for anther development by regulating tapetum development, callose dissolution and exine formation in *Arabidopsis*. Plant J..

[209] Yi J., Moon S., Lee Y.S., Zhu L., Liang W., Zhang D., Jung K.H., An G. (2016). Defective tapetum cell death 1 (DTC1) regulates ROS levels by binding to metallothionein during tapetum degeneration. Plant Physiol..

[210] Zhang D., Shi J., Yang X. (2016). Role of Lipid Metabolism in Plant Pollen Exine Development.

[211] Li Z., Zhu T., Liu S., Jiang Y., Liu H., Zhang Y., Xie K., Li J., An X., Wan X. (2021). Genome-wide analyses on transcription factors and their potential microRNA regulators involved in maize male fertility. Crop J..

[212] Wei X., Pu A., Liu Q., Leng Y., Fu Z., Wu F., An X., Long Y. (2022). Commercialization and supervision policies of gene edited crops in china and other main countries. ACS Agric. Sci. Technol..

[213] Sarić R., Nguyen V.D., Burge T., Berkowitz O., Trtílek M., Whelan J., Lewsey M.G., Čustović E. (2022). Applications of hyperspectral imaging in plant phenotyping. Trends Plant Sci..

[214] Shi Y., Thomasson J.A., Murray S.C., Pugh N.A., Rooney W.L., Shafian S., Rajan N., Rouze G., Morgan C.L., Neely H.L. (2016). Unmanned aerial vehicles for high-throughput phenotyping and agronomic research. PLoS ONE.

[215] Gupta P.K., Kulwal P.L., Jaiswal V. (2019). Association mapping in plants in the post-GWAS genomics era. Adv. Genet..

[216] Jiang Y., Sun K., An X. (2022). CRISPR/Cas system: Applications and prospects for maize improvement. ACS Agric. Sci. Technol..

