# Supplementary material for: Genetic Structure and Molecular Mechanisms Underlying the Formation of Tassel, Anther, and Pollen in the Male Inflorescence of Maize (Zea mays L.)
Source: Cells. 2022 May 26;11(11):1753. doi: 10.3390/cells11111753 (PMC9179574; doi:10.3390/cells11111753)
Supplement: Supplementary file 1 [file cells-11-01753-s001.zip › cells-1691936 Figure S1.pdf]

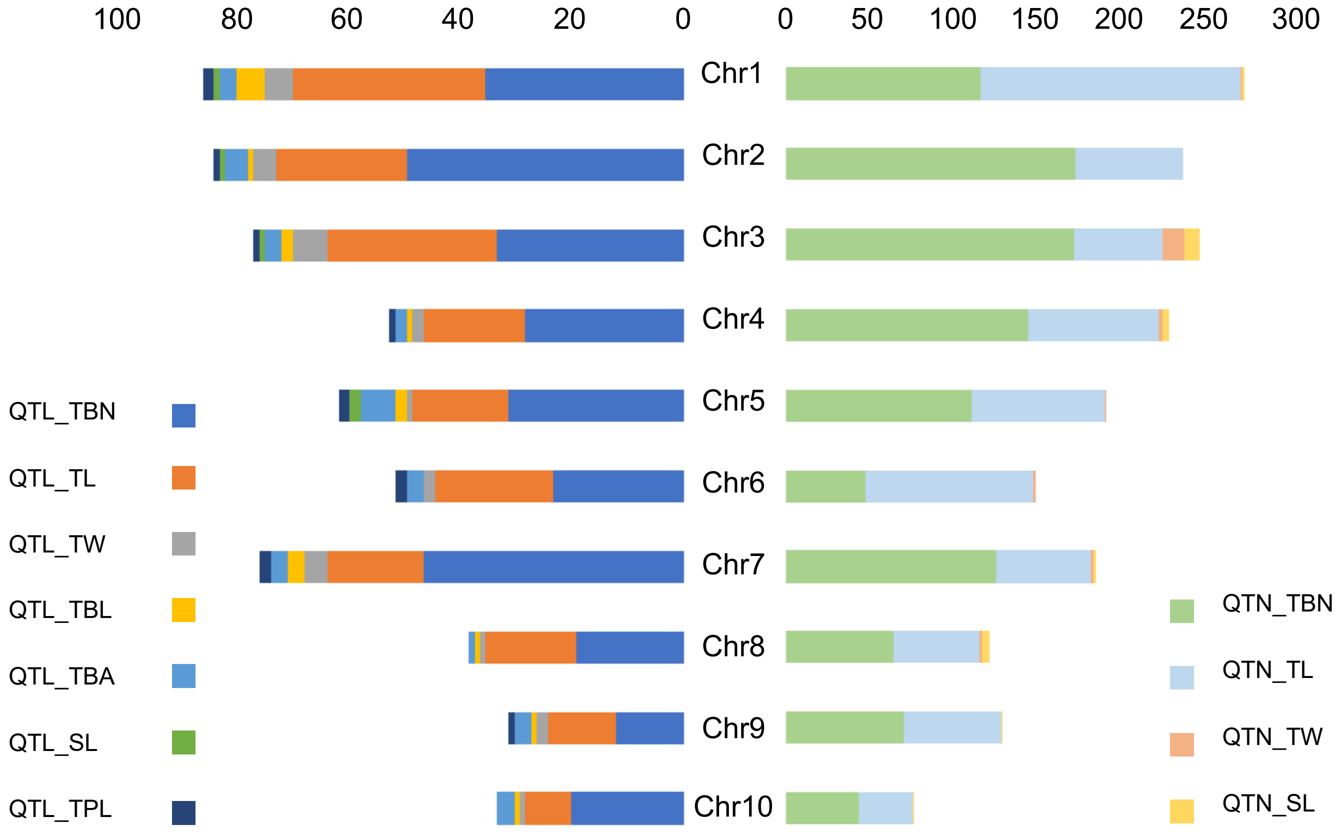

Figure S1. Summary of genetic loci for tassel-related traits on each chromosome. Two types of genetic loci (QTLs and QTNs), and seven tassel-related traits, including central spike length (SL), tassel branch angle (TBA), total branch length (TBL), tassel branch number (TBN), tassel length (TL), tassel peduncle length (TPL) and tassel weight (TW) were summarized.
